# Intrinsic Muscle Stem Cell Dysfunction Contributes to Impaired Regeneration in the *mdx* Mouse

**DOI:** 10.1002/jcsm.13682

**Published:** 2024-12-26

**Authors:** Marie E. Esper, Caroline E. Brun, Alexander Y. T. Lin, Peter Feige, Marie J. Catenacci, Marie‐Claude Sincennes, Morten Ritso, Michael A. Rudnicki

**Affiliations:** ^1^ Sprott Centre for Stem Cell Research, Regenerative Medicine Program Ottawa Hospital Research Institute Ottawa Canada; ^2^ Department of Cellular and Molecular Medicine, Faculty of Medicine University of Ottawa Ottawa Canada; ^3^ Institut NeuroMyoGène, Pathophysiology and Genetics of Neuron and Muscle, Inserm U1315, CNRS UMR5261 University Claude Bernard Lyon 1 Lyon France; ^4^ Centre Armand‐Frappier santé Biotechnologie, Institut National de la Recherche Scientifique (INRS) Unité de recherche mixte INRS‐UQAC en santé durable Laval Canada; ^5^ School of Biomedical Engineering The University of British Columbia Vancouver Canada; ^6^ Department of Medicine, Faculty of Medicine University of Ottawa Ottawa Canada

**Keywords:** Duchenne muscular dystrophy, dystrophin, *mdx*, muscle stem cell, regeneration, satellite cell

## Abstract

**Background:**

Duchenne muscular dystrophy (DMD) is a devastating disease characterized by progressive muscle wasting that leads to diminished lifespan. In addition to the inherent weakness of dystrophin‐deficient muscle, the dysfunction of resident muscle stem cells (MuSC) significantly contributes to disease progression.

**Methods:**

Using the *mdx* mouse model of DMD, we performed an in‐depth characterization of disease progression and MuSC function in dystrophin‐deficient skeletal muscle using immunohistology, isometric force measurements, transcriptomic analysis and transplantation assays. We examined the architectural and functional changes in *mdx* skeletal muscle from 13 and 52 weeks of age and following acute cardiotoxin (CTX) injury. We also studied MuSC dynamics and function under homeostatic conditions, during regeneration post‐acute injury, and following engraftment using a combination of histological and transcriptomic analyses.

**Results:**

Dystrophin‐deficient skeletal muscle undergoes progressive changes with age and delayed regeneration in response to acute injury. Muscle hypertrophy, deposition of collagen and an increase in small myofibres occur with age in the *tibialis anterior* (TA) and diaphragm muscles in *mdx* mice. Dystrophic *mdx* mouse TA muscles become hypertrophic with age, whereas diaphragm atrophy is evident in 1‐year‐old *mdx* mice. Maximum tetanic force is comparable between genotypes in the TA, but maximum specific force is reduced by up to 38% between 13 and 52 weeks in the *mdx* mouse. Following acute injury, myofibre hyperplasia and hypotrophy and delayed recovery of maximum tetanic force occur in the *mdx* TA. We also find defective MuSC polarity and reduced numbers of myocytes in *mdx* muscle following acute injury. We observed a 50% and 30% decrease in PAX7^+^ and MYOG^+^ cells, respectively, at 5 days post CTX injury (5 dpi) in the *mdx* TA. A similar decrease in *mdx* progenitor cell proportion is observed by single cell RNA sequencing of myogenic cells at 5 dpi. The global expression of commitment‐related genes is also reduced at 5 dpi. We find a 46% reduction in polarized PARD3 in *mdx* MuSCs. Finally, *mdx* MuSCs exhibit elevated PAX7^+^ cell engraftment with significantly fewer donor‐derived myonuclei in regenerated myofibres.

**Conclusions:**

Our study provides evidence that dystrophin deficiency in MuSCs and myofibres together contributes to progression of DMD. Ongoing muscle damage stimulates MuSC activation; however, aberrant intrinsic MuSC polarity and stem cell commitment deficits due to the loss of dystrophin impair muscle regeneration. Our study provides in vivo validation that dystrophin‐deficient MuSCs undergo fewer asymmetric cell divisions, instead favouring symmetric expansion.

## 1 Introduction

Duchenne muscular dystrophy (DMD) is a fatal neuromuscular disease caused by loss‐of‐function mutations in the X‐linked dystrophin gene (*DMD*). The absence of full‐length dystrophin protein (Dp427m) in both myofibres and MuSCs prevents assembly of the dystrophin‐associated glycoprotein complex (DGC), which normally tethers intracellular actin to the extracellular cytoskeleton. This renders the myofibre sarcolemma prone to contraction‐induced damage and impairs MuSC asymmetric division [1, 2].

Knowledge of DMD pathophysiology is largely based on studies in the genetically homologous *mdx* mouse. Although the progression of muscular dystrophy in *mdx* mice is less severe compared to humans, both dystrophin‐deficient mouse and human muscles display characteristic lesions, elevated inflammation and similar mechanisms of dysregulation [3]. Moreover, human and murine MuSCs express Dp427m and components of the DGC in a temporally regulated manner during myogenic commitment [2, 4].

Upon MuSC activation, the polarity kinase MARK2 interacts with Dp427m to establish the MuSC polarity required for asymmetric cell division. Without Dp427m, fewer asymmetric cell divisions occur, leading to MuSC hyperplasia and fewer progenitor cells [2]. Additionally, without assembly of the DGC, its role in asymmetrically regulating the epigenetic activation of MuSCs is perturbed, further contributing the MuSC progenitor imbalance [5]. However, the role of impaired asymmetric MuSC divisions and reduced commitment in muscle regeneration in vivo has not been extensively studied.

Here, we provide a comprehensive characterization of the physiological and morphological consequences of dystrophin deficiency in *mdx* myofibres and MuSCs during ageing up to 12 months and following acute injury. With age, we see progressive histological changes, consistently reduced normalized force and elevated pools of *mdx* MuSCs that decline in numbers over time. Upon acute cardiotoxin injury, *mdx* muscle regeneration is delayed, myofibres are hypotrophic, and we find that MuSCs generate fewer progenitors. A combination of histology and transcriptomic analysis before and following CTX injury highlights the incorrect temporal activation of MuSCs in homeostatic *mdx* muscle and an intrinsic polarity and commitment deficit in *mdx* MuSCs following activation. Analysis of neonatal myogenesis and engraftment assays further substantiate the transcriptomic data, demonstrating the intrinsically reduced regenerative capacity of *mdx* MuSCs.

## 2 Methods

Protocols, mouse strains, antibodies and reagents are detailed in the Supporting Information.

### 2.1 Mouse Models and Procedures

Experiments were performed in accordance with the University of Ottawa Animal Care Committee guidelines and approved by Animal Research Ethics Board at the University of Ottawa. Age‐ and sex‐matched C57BL/10ScSnJ (WT) and C57BL/10ScSn‐Dmd^mdx^/J (*mdx*) mice were used in this study, including mice crossed with transgenic strains. Isometric force measurements were recorded on the *tibialis anterior* (TA) muscle, as described previously [S1]. Engraftment experiments were modified from published protocols [S4]. The legs of immunodeficient NOD.Cg‐*Prkdc* ^*scid*^ *Il2rg* ^*tm1Wjl*^ */SzJ* (NSG) mice were irradiated, and the TA immediately CTX‐injured. WT and *mdx* donor MuSCs were injected into contralateral legs 48 h later.

### 2.2 Fluorescence‐Activated Cell Sorting (FACS)

Hindlimb muscles were dissociated in collagenase/dispase, stain with lineage‐negative antibodies, positive selection markers and viability dye. Antibodies are listed in Table S1.

### 2.3 Tissue Processing and Immunostaining

Muscles were perfusion fixed prior to embedding or freshly embedded in OCT and frozen in liquid nitrogen‐cooled isopentane. Muscles were transversely sectioned at 10 μm‐thickness. Perfusion fixation, processing and conventional immunostaining was performed as previously described [S5]. Primary antibodies are listed in Table S1 and other reagents in Table S2.

### 2.4 Bulk and Single‐Cell RNA‐seq

RNA‐seq library construction was performed using a polyA mRNA workflow, and scRNA‐seq libraries were generated using the 10X genomics platform. Sequenced libraries were aligned to the GRCm38 reference genome and analysed using R, with DESeq2 [S8] and Seurat [S13]. All bulk RNA‐seq and scRNA‐seq data have been deposited in GEO under accession GSE268316 (RNA‐seq: GSE268313; scRNA‐seq: GSE268314). Reviewer code: onolyyaobzwlpuf.

## 3 Results

### 3.1 Myofibre Hypertrophy, Hyperplasia and Reduced Force Generation Occur in *mdx* Muscle

To characterize the physiological consequences of dystrophin deficiency, we assessed the TA muscle of WT and *mdx* mice from 13 to 56 weeks of age. Despite the significantly lower body weight of *mdx* mice compared WT mice (Figure S1A), the *mdx* TA was heavier from 26 to 56 weeks of age (Figure S1B) [6, 7, 8].

**FIGURE 1:**
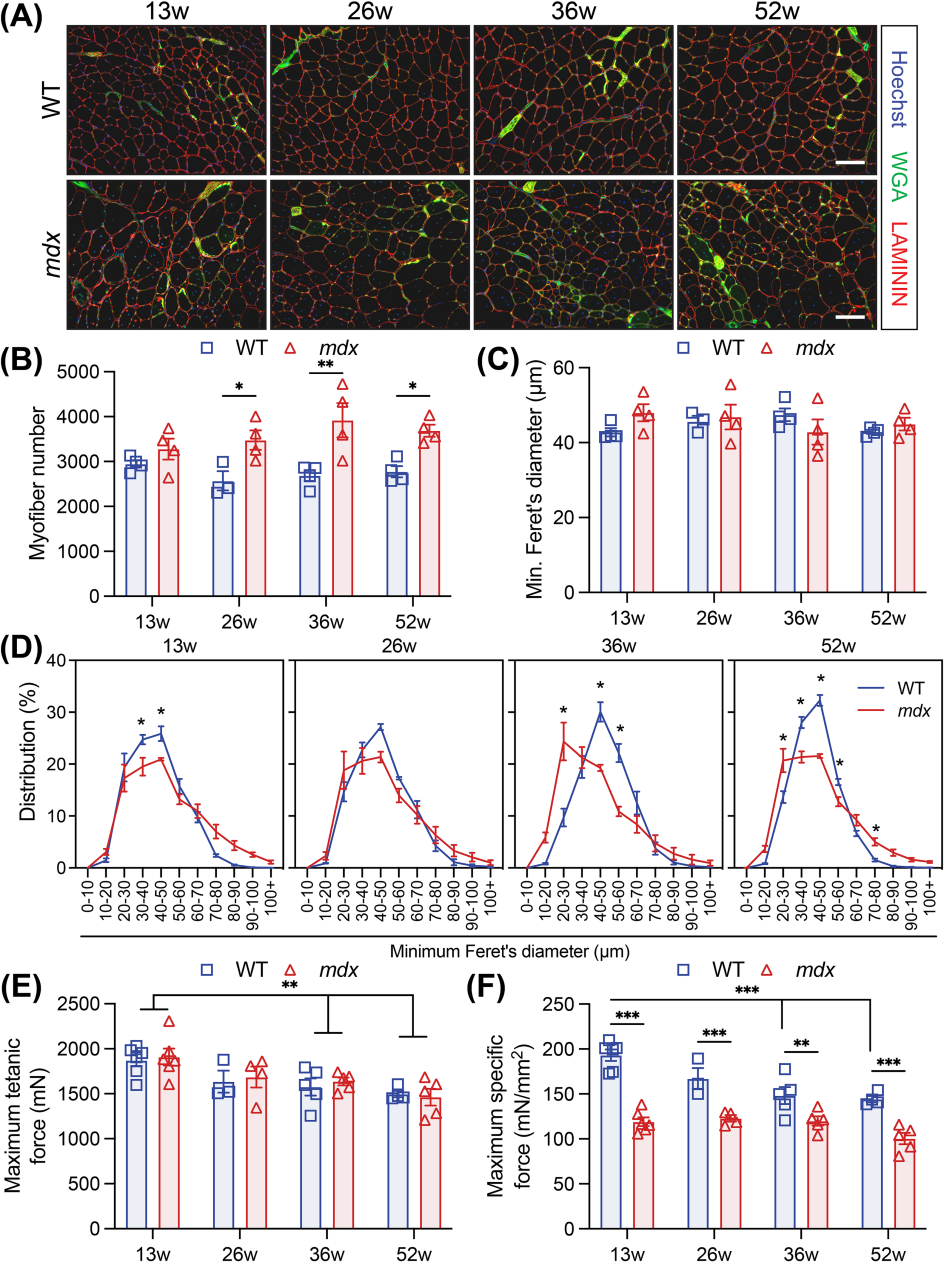
Myofibre hypertrophy, hyperplasia and reduced force generation occur in response to chronic injury in the *mdx* mouse. (A) Representative immunofluorescence images of transversal *tibialis anterior* (TA) muscle sections from 13‐, 26‐, 36‐ and 52‐week‐old wild‐type (WT) and *mdx* mice. LAMININ (red) delineates the myofibres, WGA (green) stains the connective tissues, and Hoechst (blue) labels the nuclei. (B) Quantification of the number of myofibres per transversal TA muscle sections. (C) Mean TA myofibres size using the minimum Feret's diameter. (D) Normalized TA myofibre distribution using the minimum Feret's diameter. (E) Maximum tetanic force and (F) maximum specific force of TA muscles from 13‐, 26‐, 36‐ and 52‐week‐old WT and *mdx* mice. Scale bar, 100 μm. Data presented as mean values ± SEM. For Panels B–F, two‐way ANOVA with post hoc analysis corrected for multiple comparisons using Sidak's test (*p < 0.05, **p < 0.01, ***p < 0.001). For Panels B–F, *n* = 4 WT and 4 *mdx* 13‐week‐old mice, *n* = 3 WT and 4 *mdx* 26‐week‐old mice, *n* = 4 WT and 4 *mdx* 36‐week‐old mice, *n* = 4 WT and 4 *mdx* 52‐week‐old mice. For Panels E and F, *n* = 6 WT and 6 *mdx* 13‐week‐old mice, n = 3 WT and 4 *mdx* 26‐week‐old mice, *n* = 5 WT and 5 *mdx* 36‐week‐old mice, *n* = 4 WT and 5 *mdx* 52‐week‐old mice.

Histological analysis of TA cross‐sections revealed that the number of *mdx* myofibres increased with age (Figure 1A,B). As previously observed [9], the mean diameter of TA myofibres did not change significantly from 13 to 56 weeks in WT or *mdx* mice (Figure 1C). However, the *mdx* myofibre size distribution broadened with the presence of larger myofibres and elevated numbers of small myofibres (Figures 1A,D and S1C,D) [9]. The distributional change in myofibre size with age differed for each genotype. WT myofibres increased in size from 13 to 36 weeks, but the number of large *mdx* myofibres remained unchanged, and small myofibres increased significantly from 36 to 52 weeks (Figure S1C).

To assess whether fat infiltration or fibrosis correlates with changes in *mdx* muscle function, we performed Biodipy and trichrome staining. Muscle fibrosis did not change in the WT muscle, but increased from 26 weeks in *mdx* mice (Figure S1E,F). No significant change in fat deposition occurred in either WT or *mdx* TA muscles from 13 to 36 weeks, but *mdx* muscles had a marked reduction in lipid droplets at 52 weeks compared to WT animals (Figure S1G,H).

To study the functional consequence of altered *mdx* muscle morphology, we measured in situ isometric force of the TA muscle. WT and *mdx* mice maintained comparable maximum tetanic force that declined with age (Figure 1E). However, when accounting for the TA displacement volume, *mdx* mice had significantly decreased specific force at all timepoints (Figures 1F and S1I) [7, 10].

### 3.2 Severe Fibrosis and Decreased Myofiber Diameter in the *mdx* Diaphragm

To examine the effect of chronic regeneration cycles on the *mdx* diaphragm, we assessed histological changes in the diaphragm of WT and *mdx* mice from 13 to 52 weeks. We first examined muscle hypertrophy by measuring diaphragm cross‐section width. Diaphragm thickness did not change in WT mice, whereas the *mdx* diaphragm was significantly thickened at 26 and 36 weeks. At 52 weeks, we observed diaphragm atrophy (Figure S2A–C).

**FIGURE 2:**
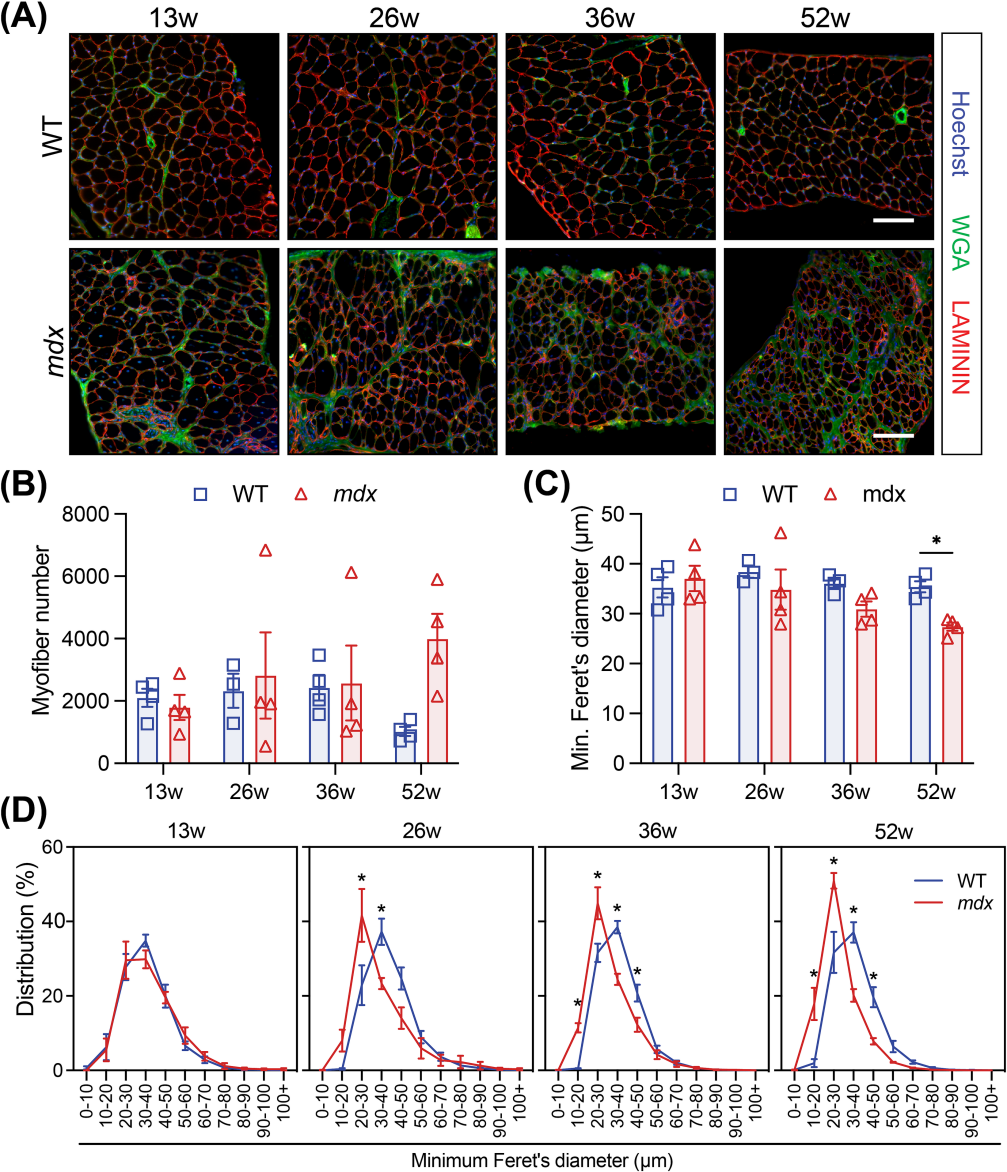
Morphology of *mdx* diaphragm becomes increasingly perturbed with age. (A) Representative immunofluorescence images of transversal diaphragm muscle sections from 13‐, 26‐, 36‐ and 52‐week‐old wild‐type (WT) and *mdx* mice. LAMININ (red) delineates the myofibres, WGA (green) stains the connective tissues, and Hoechst (blue) labels the nuclei. (B) Quantification of the number of myofibres per quantified diaphragm section. (C) Mean diaphragm myofibre size using the minimum Feret's diameter. (D) Normalized TA myofibre size distribution using minimum Feret's diameter. Scale bar, 100 μm. Data presented as mean values ± SEM. Two‐way ANOVA corrected for multiple comparisons using Sidak's test (*p < 0.05). *n* = 4 WT and 4 *mdx* 13‐week‐old mice, *n* = 3 WT and 4 *mdx* 26‐week‐old mice, *n* = 4 WT and 4 *mdx* 36‐week‐old mice, *n* = 4 WT and 4 *mdx* 52‐week‐old mice.

The number of myofibres in the WT and *mdx* diaphragm could not be directly compared due to variability in cross‐sectional coverage (Figure 2A,B). We therefore assessed the minimum myofibre diameter and the proportional change in myofibre size distribution, which did not change with age in the WT diaphragm (Figures 2C,D and S2B). In the *mdx* diaphragm, the minimum myofibre diameter decreased linearly with age (Figure 2A,C) due to a dramatic increase in small myofibres and the concurrent loss of large myofibres from 26 weeks of age (Figures 2A,D and S2B). Myofibre hypertrophy was not observed in the *mdx* diaphragm (Figures 2A,D and S2B,C).

Trichrome staining revealed a progressive increase (up to 9.5‐fold) in interstitial collagen deposition in the diaphragm of *mdx* mice, representing up to 51% of total diaphragm area (Figure S2D,E). A variable decrease in lipid deposition within the diaphragm of *mdx* mice was observed; however, this accounted for < 1% of the muscle (Figure S2F).

### 3.3 Delayed Recovery Following Acute Injury Is Caused by Myofiber Hyperplasia in *mdx* Muscle

To study muscle regeneration following acute injury, we injected CTX into the TA of WT and *mdx* mice. Within genotypes, CTX injury did not result in changes to the body weight of WT or *mdx* mice (Figure S3A). However, decreased TA mass due to myolysis was observed at 5 dpi, after which the muscle weight increased (Figure S3B). By 21 dpi, the WT TA recovered its pre‐injury mass, whereas the *mdx* TA weight increased by 62% compared to uninjured muscle (Figure S3B) [11]. Between 21 and 90 dpi, the WT and *mdx* TA weight increased by 40% and 20%, respectively (Figure S3B). However, the *mdx* TA weighed significantly more than the control WT TA at 21 and 90 dpi (Figure S3B).

**FIGURE 3:**
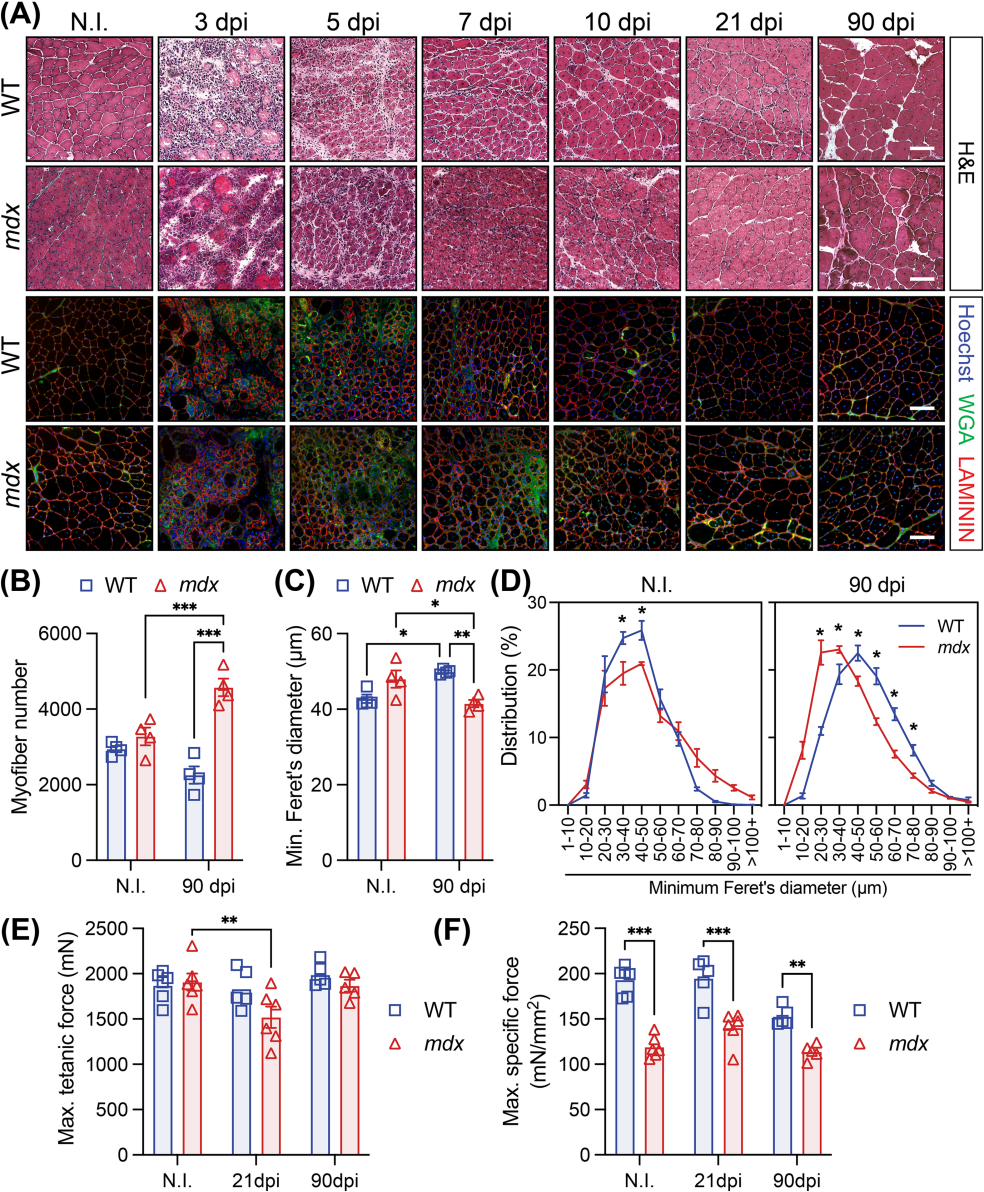
Delayed regeneration of *mdx* muscle following acute injury. (A) Representative haematoxylin and eosin (H&E) and immunofluorescence images of transversal sections of non‐injured (NI) and cardiotoxin (CTX)‐injured *tibialis anterior* (TA) muscle at 3, 5, 7, 10, 21 and 90 days post‐injury (dpi) from wild‐type (WT) and *mdx* mice. LAMININ (red) delineates the myofibres, WGA (green) stains the connective tissues and Hoechst (blue) labels the nuclei. (B) Cross‐sectional TA myofibre quantification at 90 dpi compared to NI. (C) Mean WT and *mdx* TA myofibre size using the minimum Feret's diameter in NI and CTX‐injured muscles at 90 dpi. (D) Normalized TA myofibre size distribution using the minimum Feret's diameter. (E) Maximum tetanic force of TA muscle from NI, 21 and 90 dpi WT and *mdx* mice. (F) Maximum specific force of TA muscles from NI, 21 and 90 dpi WT and *mdx* mice. Scale bar, 100 μm. Data presented as mean values ± SEM. Two‐way ANOVA corrected for multiple comparisons using Sidak's test (*p < 0.05; **p < 0.01; ***p < 0.001). NI = non‐injured. For Panels B–D, *n* = 4 WT and 4 *mdx* ‘non‐injured’ mice, *n* = 5 WT and 5 *mdx* ‘90 dpi’ mice. For Panels E and F, *n* = 6 WT and 6 *mdx* ‘non‐injured’ mice, *n* = 5 WT and 6 *mdx* ‘21 dpi’ mice, *n* = 5 WT and 5 *mdx* ‘90 dpi’ mice.

The morphology of regenerating WT and *mdx* muscle was visually comparable up to 90 dpi, but differences were observed (Figures 3A–C and S3C). Compared to non‐injured (NI) muscle, the number of WT myofibres remained unchanged at 90 dpi, but the mean minimum myofibre diameter increased (Figure 3B,C). By contrast, the number of small *mdx* myofibres increased dramatically, and myofibre hypertrophy was not observed (Figures 3B,D and S3E,F). The *mdx* TA histopathology worsened after triple CTX injury (3 × 21 dpi; 3X CTX). In the *mdx* TA muscle, the number of myofibres increased, and the minimum myofibre diameter decreased. Conversely, the WT TA morphology was unaffected (Figure S4A–C). Collagen deposition was also not significantly changed in the WT or *mdx* TA at 90 dpi or after triple injury (Figures S3D and S4D,E). Together, this suggests that muscle growth following acute injury occurs by myofibre hypertrophy in WT muscle and myofibre hyperplasia in *mdx* muscle.

To determine the physiological consequences of architectural changes following acute injury, we measured in situ isometric force of the WT and *mdx* TA at 21 and 90 dpi. Maximum titanic force was recovered by 21 dpi in WT mice but not until 90 dpi in the *mdx* TA (Figure 3E). Specific force was significantly reduced at both 21 and 90 dpi (Figures 3F and S3G). Following triple injury, the maximum tetanic force increased in the WT TA but was unchanged in the *mdx* TA compared to NI conditions. Similarly, the maximum tetanic force of the *mdx* TA was reduced compared to the WT muscle (Figure S4F).

### 3.4 Perturbed Muscle Stem Cell Homeostasis and Injury Response

**FIGURE 4:**
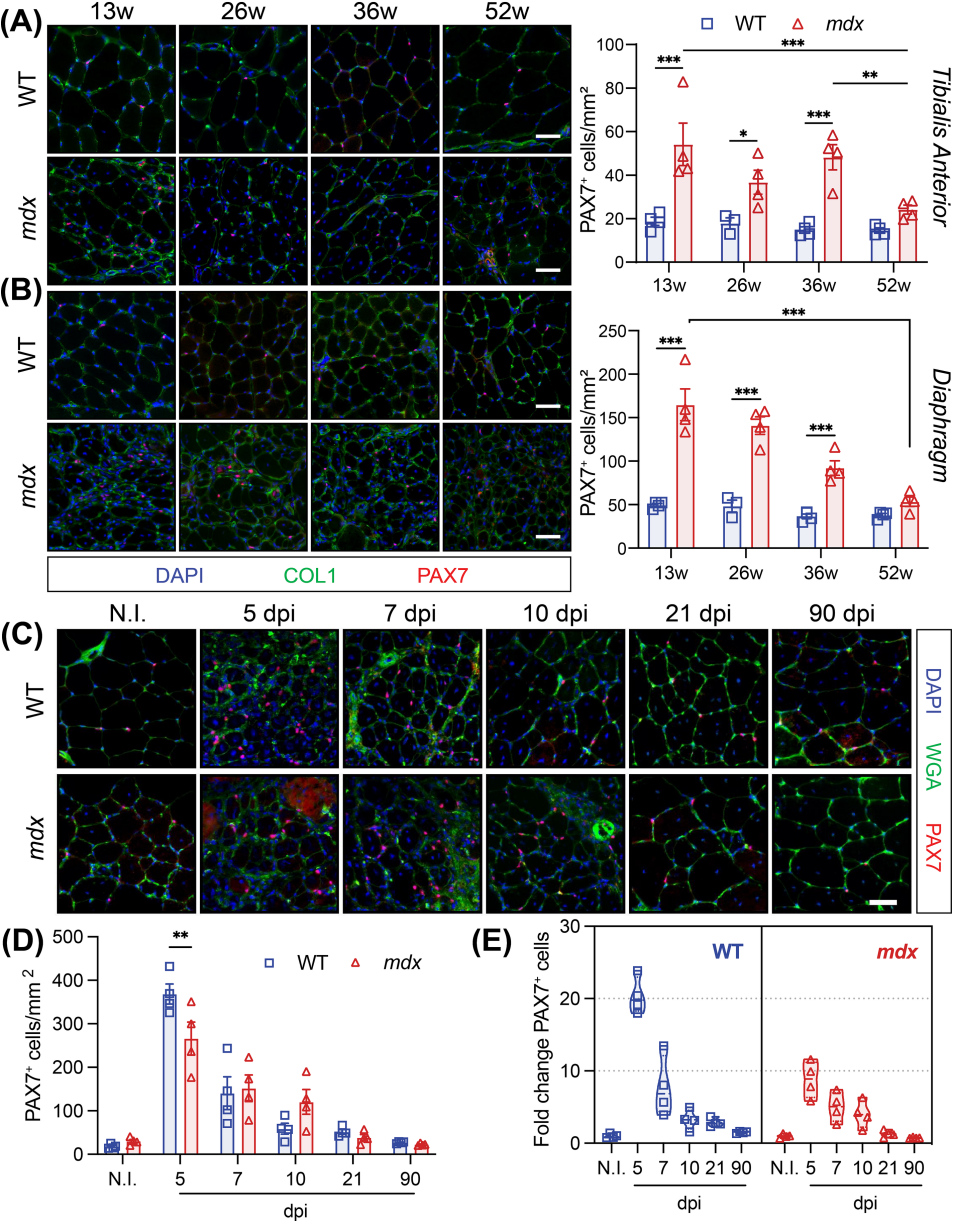
Perturbed *mdx* MuSC homeostasis and activation. (A,B) Immunofluorescence labelling and quantification of PAX7‐expressing muscle stem cells (MuSCs, red) on WT and *mdx tibialis anterior* (TA, top, Panel A) and diaphragm (bottom, Panel B) muscle cross‐sections. Collagen1 (COL1, green) labels the extracellular matrix and Hoechst (blue) labels the nuclei. For Panels A and B, n = 4 WT and 4 *mdx* 13‐week‐old mice, n = 3 WT and 4 *mdx* 26‐week‐old mice, n = 4 WT and 4 *mdx* 36‐week‐old mice, n = 4 WT and 4 *mdx* 52‐week‐old mice. (C) Representative immunofluorescence images of transversal sections from WT and *mdx* non‐injured (N.I.) and cardiotoxin (CTX)‐injured TA muscles at 5, 7, 10, 21 and 90 days post‐injury (dpi) from wild‐type (WT) and *mdx* mice. PAX7 (red) marks the muscle stem cells (MuSCs), WGA (green) stains the connective tissues, and Hoechst (blue) labels the nuclei. (D) PAX7‐expressing MuSC number per mm^2^ TA sections at the corresponding days following injury. *n* = 4 mice/genotype. (E) Fold change of PAX7^+^ MuSCs at each timepoint post CTX‐injury relative to no‐injury (N.I.) conditions. *n* = 4 mice/genotype. Two‐way ANOVA corrected for multiple comparisons using Sidak's test (*p < 0.05; **p < 0.01; ***p < 0.001). Scale bar, 100 μm. Data presented as mean values ± SEM.

To investigate the dynamics of *mdx* MuSCs in response to chronic repeat injury, we compared the number of PAX7‐expressing (PAX7^+^) cells in WT and *mdx* muscle from 13 to 56 weeks of age. Consistent with previous reports [12, 13], the number of PAX7^+^ cells was elevated in the *mdx* TA and diaphragm at all timepoints (Figure 4A,B). However, whereas the number of PAX7^+^ cells did not change in the WT TA or diaphragm, the number of *mdx* MuSCs declined in both muscles between 13 and 52 weeks (Figure 4A,B).

We next assessed the response of PAX7^+^ MuSCs to acute CTX injury by immunostaining a regeneration time course of WT and *mdx* TA cross‐sections (Figure 4C). Significant expansion of WT and *mdx* PAX7^+^ cells occurred at 5 dpi, followed by a progressive decrease to NI levels at 21 dpi (Figure 4D). However, fewer *mdx* PAX7^+^ were enumerated at 5 dpi (Figure 4D). We also normalized the number of PAX7^+^ cells at 5 dpi to uninjured conditions within both genotypes and observed reduced expansion in *mdx* PAX7^+^ cells (8‐fold) relative to WT cells (20‐fold) at 5 dpi (Figure 4E). Interestingly, the number of PAX7^+^ cells increased in the WT TA muscle following triple CTX‐induced injury. Inversely, fewer *mdx* PAX7^+^ cells were quantified following repeat injury (Figure S4G,H).

### 3.5 Dystrophin‐Deficient MuSCs Display Features of Activation in *mdx* Muscle

To characterize the impact of Dp427m‐deficiency on MuSCs in homeostatic and injured muscle, we studied the transcriptomic profile of WT and *mdx* MuSCs by bulk and single‐cell RNA sequencing (RNA‐seq) at multiple stages in the myogenic differentiation trajectory.

Bulk RNA‐seq was performed in biological triplicates on myogenic cells prospectively isolated from WT and *mdx* mice before injury and at 3 dpi, termed freshly isolated (Fi)MuSC and activated (A)MuSCs, and from cultured primary myoblasts and 2‐day differentiated myotubes (Figure S5A–C). Principal component analysis (PCA) of the bulk RNA‐seq libraries revealed that cell state is a primary driver of variance (Figure S5C,D). Although the FiMuSC and 2‐day differentiated myotube grouped by myogenic state regardless of genotype, greater variance between genotypes was observed in the AMuSC and myoblast libraries. We next performed differential gene expression testing between genotypes for all conditions (Data S1).

**FIGURE 5:**
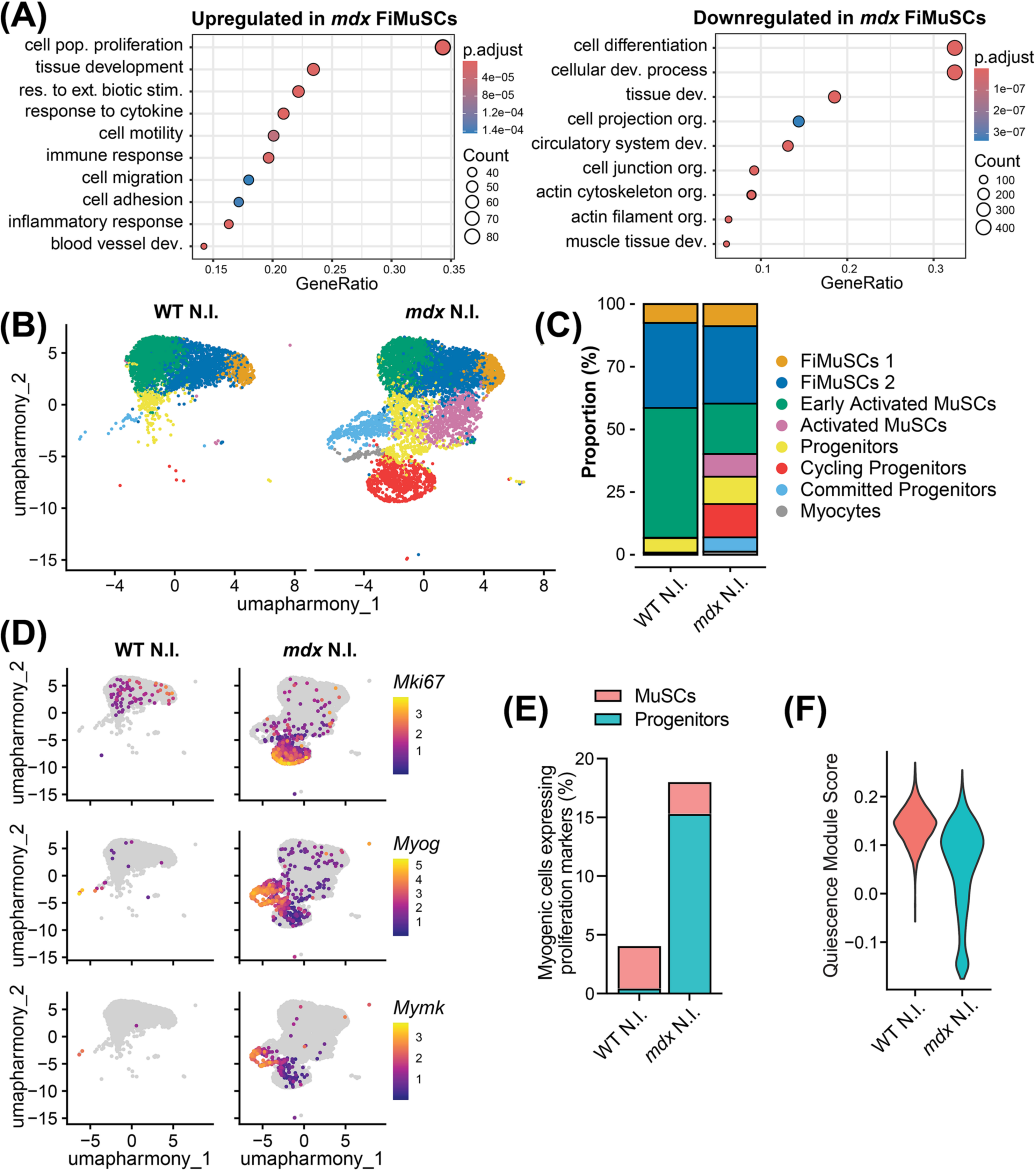
Increased activation of *mdx* MuSCs in homeostatic muscle. (A) Gene ontology term enrichment for the upregulated (left) and downregulated (right) genes (*p*_*adjusted*_ < 0.05, Log2FC > 2) in *mdx* compared to wild‐type (WT) freshly isolated muscle stem cells (FiMuSCs). (B) Uniform manifold approximation and projection (UMAP) visualization of cells (4205 WT and 6980 *mdx*) captured from uninjured MuSC libraries and coloured by cluster identity. (C) Bar plot representing cluster proportions for uninjured WT and *mdx* MuSC libraries. (D) Feature plot illustrating the expression of proliferation marker *Mki67* and commitment markers *Myog* and *Mymk*. (E) Proportion of myogenic cells expressing *Cenpa*, *Mki67* or *Cdk1*, split by MuSC and progenitor cell identity. (F) Pseudo bulk quiescence module score (top 400 enriched from Garcia‐Prat et al. [14]) divided between uninjured WT and *mdx* libraries.

Differential gene expression (DGE) analysis between the WT and *mdx* FiMuSC libraries identified 1089 upregulated and 613 downregulated genes in *mdx* FiMuSCs (Figure S5E and Data S1). Gene ontology (GO) analysis of the upregulated genes in the *mdx* FiMuSC library identified terms including ‘cell population proliferation’, ‘tissue development’, ‘response to external stimulus’ and ‘cell motility’ (Figure 5A and Data S2), indicating that *mdx* MuSCs are not completely quiescent. Further, terms related to the immune response were identified that reflect the inflammatory milieu surrounding *mdx* MuSCs. GO analysis on the downregulated genes in the *mdx* FiMuSC library highlighted terms such as ‘cell differentiation’ and ‘muscle tissue development’, suggesting that despite increased proliferation *mdx* MuSCs have reduced commitment capacities (Figure 5A).

Four single‐cell RNA‐seq (scRNA‐seq) libraries were also generated from MuSCs isolated from NI and 5 dpi WT and *mdx* hindlimb muscle (Figure S6A). After integration, ten clusters were identified and annotated based on differential gene expression and known cell markers (Figure S6B–E and Data S3). Three contaminating clusters, namely, *Cdh5* ^+^ ‘Endothelial Cells’, *Ckm* ^+^ ‘Myonuclei’ and *Ptprc* ^+^ ‘Immune Cells’, were equally distributed in all four libraries (1.5% and 2.4% of the WT and *mdx* cells, respectively) and were excluded from downstream analysis (Figure S6B,C).

The seven myogenic clusters encompassed the trajectory from quiescent MuSCs to myocytes. By assigning a quiescence module score to individual cells based on previously published ‘quiescence core signature’ genes, we identified three MuSC clusters with higher quiescence signatures (Figure S6F) [14], termed freshly isolated MuSCs 1 (‘FiMuSCs 1’), ‘FiMuSCs 2’ and ‘Early Activated MuSCs’ (Figure S6B,D). Elevated expression of *Pax7*, *Calcr* and oxidative phosphorylation (OxPhos) genes [15] together with the absence of myogenic regulatory factor (MRF) gene expression suggested FiMuSCs 1 was the most quiescent (Figure S6D,E). Early Activated MuSCs were enriched in early activation and stress response–associated genes that are induced upon tissue dissociation, including *Jun*, *Junb* and *Fos* (Figure S6B,D,E) [16]. The *Pax7* ^+^ ‘Activated MuSC’ cluster, identified by reduced expression of quiescence genes and extracellular matrix gene enrichment, expressed negligible markers of myogenic cell proliferation, including *Mki67, Cenpa* and *Myod1* (Figure S6D). Similarly, Seurat's CellCycleScoring function categorized less than 15% of Activated MuSCs having entered mitosis, which was consistent with the FiMuSCs 1, FiMuSC2 and Early Activated MuSCs clusters. In contrast, two myogenic progenitor clusters were identified. ‘Progenitor’ and ‘Cycling Progenitor’ expressed elevated *Myf5* and *Myod1*, whereas the latter was enriched for S/G2/M‐associated genes, including *Mki67* and *Cenpa*. ‘Committed Progenitors’ and ‘Myocytes’ were largely post‐mitotic (*Cdkn1c* ^+^) and expressed *Myog*. ‘Myocytes’ expressed elevated levels of commitment markers, including *Mymk* and *Acta1* (Figure S6D).

To investigate the intrinsic state and activation of dystrophic MuSCs, we assessed the scRNA‐seq libraries isolated from WT and *mdx* uninjured muscle. FiMuSCs 1, FiMuSCs 2 and Early Activated MuSCs accounted for 93% and 59% of the uninjured WT and *mdx* libraries, respectively. The *mdx* library instead consisted of cells across the entire myogenic continuum, from *Pax7* ^+^ quiescent MuSCs to cycling progenitors and terminally differentiating myocytes (Figure 5B,C). Further, markers of proliferation and differentiation were expressed in the *mdx* but negligibly in the WT uninjured libraries (Figure 5D).

To examine the proportion of actively proliferating MuSCs in homeostatic muscle, we first regressed the Cell Cycle score from the Seurat object using the scale.data function to ensure that cell cycle was not the primary driver of clustering. We then examined the proportion of myogenic cells from the uninjured libraries that expressed any of the cell cycle markers *Cenpa*, *Mki67* and *Cdk1*. We found that 4% of WT and 18% of *mdx* myogenic cells were actively proliferating (Figure 5E). Within this analysis, we divided cells into two groups, categorizing the four stem cell clusters as ‘MuSCs’ and the remaining clusters as ‘progenitors.’ Notably, the number of proliferating MuSCs was comparable between genotypes, whereas the number of proliferating *mdx* progenitors increased. Moreover, pseudo‐bulk analysis of the uninjured libraries revealed an overall lower quiescence module score in *mdx* myogenic cells (Figure 5F) [14]. Together, this suggests that dystrophin‐deficient MuSCs from homeostatic muscle are in an asynchronous state of activation, leading to increased numbers of progenitor cells and differentiation [12, 17]. Importantly, WT MuSCs are normally quiescent in uninjured muscle, whereas *mdx* MuSCs are pathogenically activated. Therefore, the presence of progenitors in the uninjured *mdx* libraries does not reflect the relative activation ability of *mdx* MuSCs compared to WT.

### 3.6 Reduced Commitment of *mdx* Muscle Stem Cells

The cyclic muscle degeneration and regeneration that results from myofibre fragility in *mdx* mice is not comparable to the basal level of muscle turnover that occurs in healthy muscle. Therefore, to compare the regenerative capacity of WT and *mdx* MuSCs, we examined bulk and single‐cell RNA‐seq MuSC libraries isolated from CTX‐injured muscle.

**FIGURE 6:**
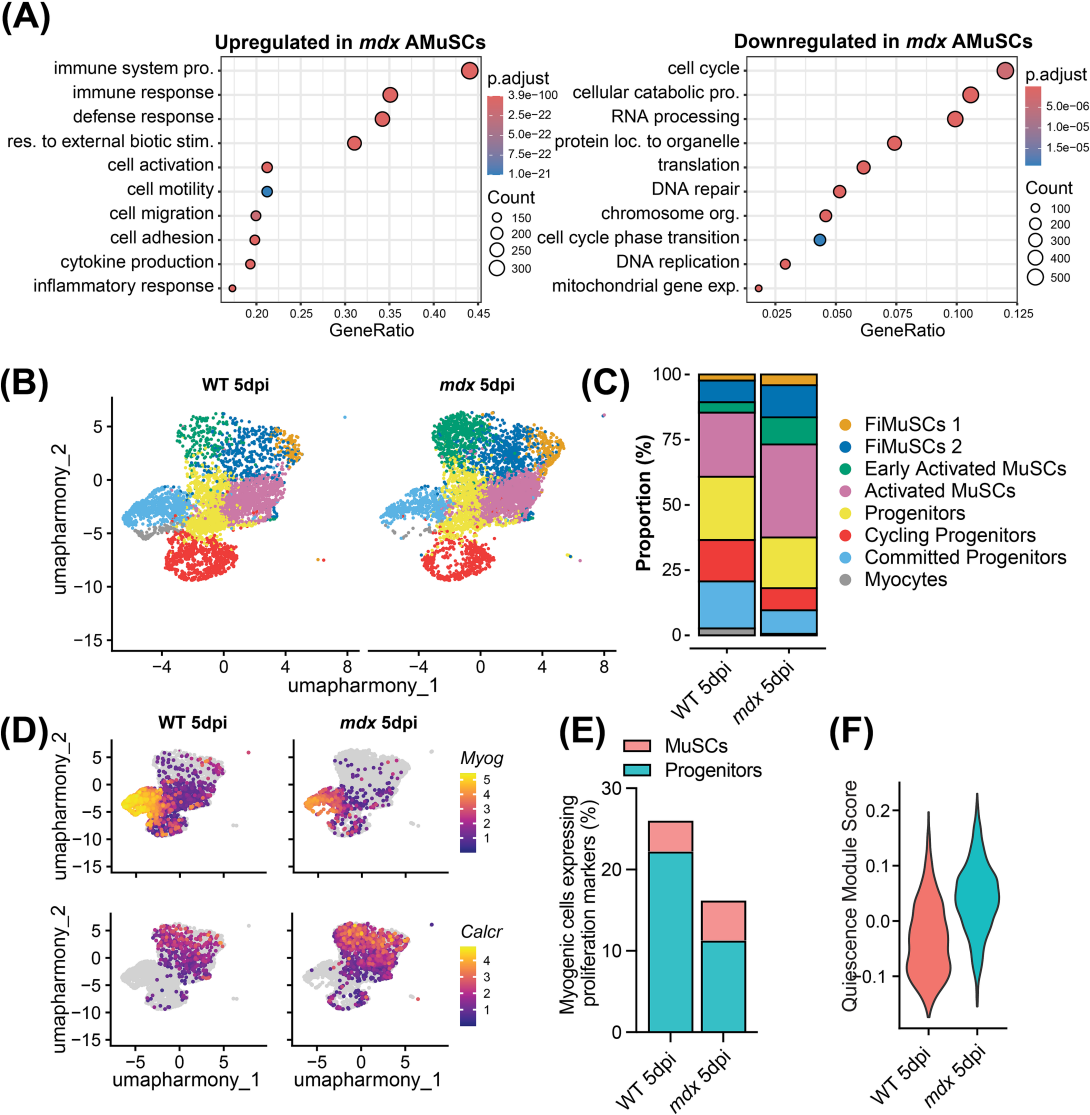
Reduced generation of *mdx* progenitor cells following acute injury. (A) Gene ontology term enrichment for the upregulated (left) and downregulated (right) genes in *mdx* compared to wild type (WT) activated muscle stem cells (AMuSCs). (B) Uniform manifold approximation and projection (UMAP) visualization of cells (4616 WT and 5396 *mdx*) captured from 5‐day cardiotoxin injured (5 dpi) MuSC libraries and coloured by cluster identity. (C) Bar plot representing cluster proportions for 5‐day injured WT and *mdx* MuSC libraries. (D) Feature plot illustrating the expression of commitment markers *Myog* and *Mymk* and quiescence marker *Calcr*. (E) Proportion of myogenic cells expressing *Cenpa*, *Mki67* or *Cdk1*, divided by MuSC and progenitor cell identity. (F) Pseudo bulk quiescence module score (top 400 enriched from Garcia‐Prat et al. [14]) divided between 5 dpi WT and *mdx* libraries.

PCA analysis of the bulk RNA‐seq libraries revealed that *mdx* AMuSC were transcriptionally more like WT FiMuSCs, whereas WT AMuSCs were more like in vitro cultured myoblasts than FiMuSCs (Figure S5C,D). DGE assessment between WT and *mdx* AMuSCs identified 2558 upregulated and 3415 downregulated genes (Figure S5F and Data S1). GO term analysis showed *mdx* AMuSCs have decreased expression of genes involved in ‘cell cycle’, ‘cell catabolic process’ and ‘RNA processing’ while maintaining increased gene expression related to ‘inflammatory response’ and ‘cell activation’ (Figure 6A and Data S2).

To study progenitor dynamics and the granularity of individual cell behaviour following MuSC activation, we next examined the scRNA‐seq libraries generated from WT and *mdx* MuSCs at 5 dpi (Figure S6A). The WT and *mdx* libraries encompassed the myogenic trajectory from MuSCs to myocytes, but the proportion of uncommitted MuSCs relative to the total number of myogenic cells was elevated in the *mdx* library. Specifically, 40% of the WT compared to 63% of the *mdx* library were categorized as either FiMuSCs 1, FiMuSCs 2 or Early Activated MuSCs. The remaining 60% and 37% of cells clustered as progenitor cells or myocytes (Figure 6B,C). Moreover, the activated WT libraries expressed elevated differentiation markers including *Myog* and reduced stemness markers, such as *Calcr* (Figure 6D). The pseudo‐bulked 5‐dpi *mdx* library also expressed elevated levels of quiescence genes compared to the WT library (Figure 6F). These results suggest that *mdx* MuSCs have fewer differentiation competent progenitors, relative to WT MuSCs.

Interestingly, 26% of WT and 16% of *mdx* myogenic cells expressed one of the proliferation markers *Cenpa*, *Mki67* and *Cdk1* following Cell Cycle regression (Figure 6E). We again divided the myogenic cells into two categories, ‘MuSCs’ and ‘progenitors,’ and found that the proportion of proliferating MuSC was unchanged between genotypes, whereas the proportion of proliferating progenitors decreased in the *mdx* library (Figure 6E). This suggests that the reduced number of PAX7^+^ cells quantified on *mdx* TA cross‐sections at 5 dpi (Figure 4C–E) results from impaired generation of progenitors, and not from differences in the proliferation kinetics of MuSCs.

We previously discovered that the direct loss of MARK2–DMD interactions in MuSC cultured on myofibres results in the loss of PARD3 polarity, fewer asymmetric cell division and reduced numbers of committed myogenin (MYOG)‐expressing cells [2, 5]. To investigate *mdx* MuSC polarity in vivo, we isolated WT and *mdx* MuSCs 48 h following CTX injury and examined polarization of the PAR complex protein PARD3. Strikingly, we observed a significant reduction in the number of *mdx* MuSCs with polarized PARD3 expression (Figure S7A).

To determine if impaired MuSC polarity leads to fewer committed progenitors in vivo, as suggested by our transcriptomic analysis, we quantified MYOG^+^ cells on TA muscle cross‐sections at 5 dpi. A twofold decrease in MYOG^+^ cells was observed on *mdx* muscle cross‐sections (Figure S7B,C). To account for the reduced number of *mdx* PAX7^+^ cells at 5 dpi that may generate fewer MYOG^+^ progeny, we also calculated the proportion of MYOG^+^ cells within the total PAX7^+^ and MYOG^+^ myogenic population. We observed a decreased proportion of *mdx* differentiated cells compared to WT (Figure S7D). Finally, we observed fewer GFP^+^ nuclei on TA cross‐sections from WT and *mdx Myf5cre;ROSA‐nTnG* mice at 7 dpi, which includes all primed MuSCs, progenitors and myonuclei (Figure S7E,F). Together, our results support the finding that *mdx* MuSCs have intrinsic polarity deficits that lead to fewer asymmetric divisions and reduced MYOG^+^ progenitor generation [2, 5].

### 3.7 Intrinsic MuSC Dysfunction Impairs Myogenesis

**FIGURE 7:**
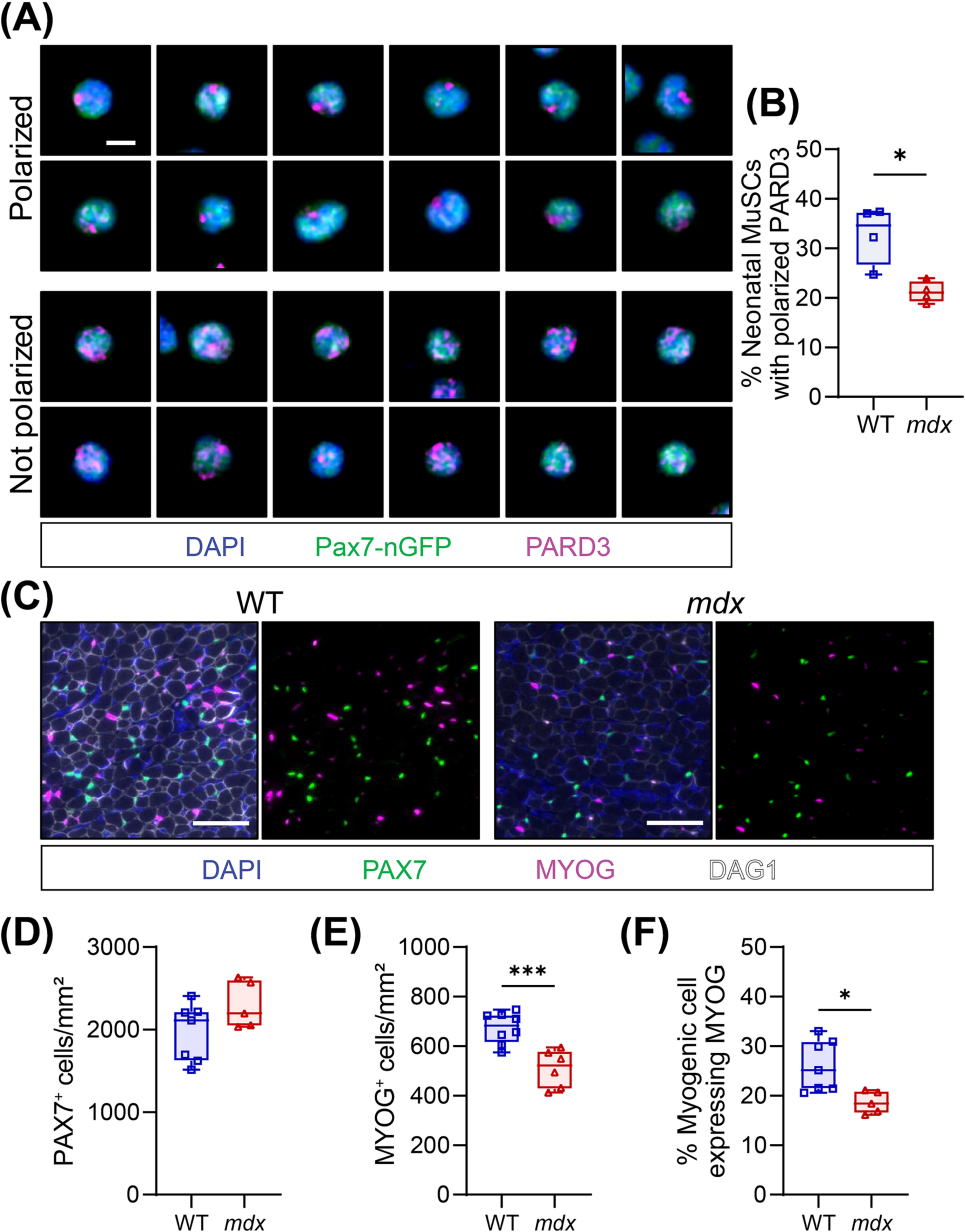
Reduced polarity and commitment of neonatal MuSCs. (A) Representative immunostaining of polarized and non‐polarized PARD3 (magenta) in MuSCs (Pax7‐nGFP, green) isolated from the limbs of neonatal day 7 mice. Scale bar, 5 μm. (B) Quantification of PARD3 polarization in neonatal MuSCs. An average of 536 cells quantified per replicate. (C) Example immunofluorescence image of neonatal day 7 WT and *mdx* hindlimb muscle transverse section. PAX7 (green) denotes the neonatal MuSCs, MYOG (magenta) stains the differentiated myogenic cells, DAG1 (white) labels the connective tissues, and DAPI (blue) labels the nuclei. Scale bar, 50 μm. (D,E) Enumeration of PAX7‐expressing (PAX7^+^) (D) and MYOG‐expressing (MYOG^+^) (E) cells on *extensor digitorum longus* (EDL) and *tibialis anterior* (TA) cross‐sections at neonatal day 7 and normalized to mm^2^. (F) MYOG^+^ cell proportion relative to total PAX7^+^ and MYOG^+^ myogenic cells at neonatal day 7. Boxplot whiskers represent the maximum and minimum data values. Statistical analysis performed using unpaired *t*‐tests where *p < 0.05; **p < 0.01; ***p < 0.001.

To assess whether *mdx* MuSCs are intrinsically dysfunctional, we first analysed MuSC polarity and myogenic cell commitment at perinatal day 7 (P7), before muscle weakness induces cyclic regeneration, chronic inflammation and severe necrosis [3]. Interestingly, disrupted PARD3 polarity was observed in P7 MuSC isolated from *mdx* limbs (Figure 7A,B). Further, *extensor digitorum longus* (EDL) and TA muscle cross‐sectional analysis revealed no difference in the number of PAX7^+^ cells between genotypes (Figure 7C,D). However, *mdx* neonatal muscle contained fewer MYOG^+^ cells, and the proportion of MYOG^+^ cells in the total PAX7^+^ and MYOG^+^ cell population was reduced (Figure 7E,F). This interestingly correlates with hypotrophic *mdx* neonatal myofibres [18].

**FIGURE 8:**
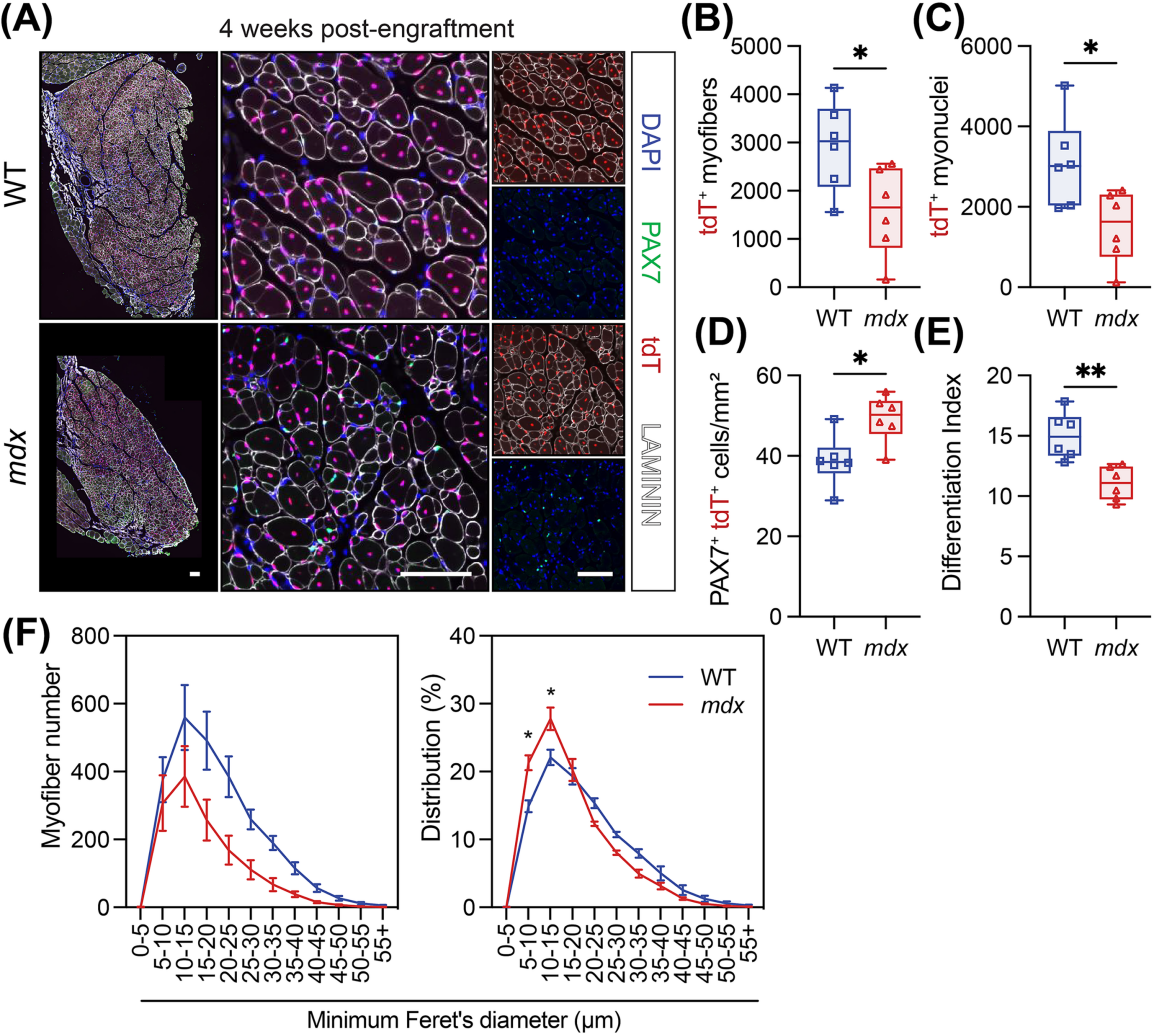
Intrinsic MuSC dysfunction impairs muscle regeneration. (A) Immunostaining of host *tibialis anterior* (TA) muscle cross‐sections 4 weeks following the engraftment of 10 000 MuSCs isolated from *WT;ROSA‐nTnG* or *mdx; ROSA‐nTnG* mice. Donor MuSCs (PAX7, green) and myonuclei express nuclear tdTomato (tdT, red). Laminin (white) stains myofibre basal lamina and DAPI (blue) stains the nuclei. Scale bars: left panels, 250 μm; middle and right panels, 100 μm. (B) Number of tdT^+^ myofibres per TA muscle transverse section. (C) Number of tdT^+^ myonuclei per TA cross‐section . (D) PAX7^+^/tdT^+^ MuSC number normalized to mm^2^ of transplanted muscle cross‐sections. (E) Differentiation index is the ratio of tdT^+^ myonuclei to tdT^+^ MuSCs. (F) Myofibre size distribution using minimum Feret's diameter and illustrated by number of myofibres (left) and proportional distribution (right). For B‐E, box plot whiskers indicate the maximum and minimum data values. For F, data presented as mean values ± SEM. Panels B–E: Statistical analysis performed using unpaired *t*‐tests. Panel F: Two‐way ANOVA corrected for multiple comparisons using Sidak's test. *p < 0.05; **p < 0.01; ***p < 0.001.

Next, we conducted transplantation experiments to remove the external contributions of the dystrophic environment. 10 000 MuSCs isolated from WT and *mdx* fluorescent reporter mice were injected contralaterally into the TAs of NSG mice 2 days following CTX injury and irradiation. The engraftment potential of WT and *mdx* MuSCs was assessed at 2 and 4 weeks following the transplantation of donor MuSCs from *CAG‐GFP* (GFP^+^) mice and *ROSA‐nTnG* (tdTomato or tdT^+^) mice, respectively (Figure S8A). The absence of dystrophin staining on *mdx* transplants at 2 and 4 weeks post engraftment confirmed negligible contribution of host NSG MuSCs to donor grafts (Figure S8B,C). This was further confirmed by the colocalization of nuclear tdT with the majority of myonuclei and PAX7^+^ cells at 4 weeks following MuSC delivery (Figure 8A).

MuSCs from both genotypes were capable of self‐renewal and differentiation [19], shown by the presence of PAX7^+^ cells and engrafted myofibres (Figures 8A and S8B). The total number of engrafted WT and *mdx* PAX7^+^ cells was also not significantly different (Figure S8D). However, *mdx* donor MuSCs formed significantly smaller transplants with fewer myofibres and myonuclei compared to WT MuSCs (Figures 8B,C and S8E,F). The number of *mdx* PAX7^+^ donor cells normalized to graft area in mm^2^ was also elevated at 2 and 4 weeks post engraftment (Figures 8D and S8G), and the ratio of myonuclei to MuSCs (differentiation index) was significantly decreased in the 4‐week *mdx* graft (Figure 8E). Further, myofibre size distribution analysis demonstrated that engrafted *mdx* MuSC form smaller myofibres 4 weeks following engraftment (Figure 8F). Together, this suggests that *mdx* MuSCs display enhanced self‐renewal together with a compromised capacity to generate the progenitors required to form myofibres.

## 4 Discussion

Our study of chronic and acute muscle regeneration in the *mdx* mouse provides a comprehensive overview of muscular dystrophy progression and contributes valuable insight into the altered dynamics and function of dystrophin‐deficient MuSCs. We find that the pathology of DMD is driven not only by myofibre fragility and the resulting inflammatory environment but also intrinsic polarity deficits that result in fewer asymmetric divisions.

Consistent with the literature, we find that Dp427m is essential for myofibre integrity and that the disease pathology worsens with age [7, 10]. We observe hallmark pathological features of dystrophin‐deficient muscle, including elevated mass and thickening, increased fibrosis and changes in myofibre size distribution [9, 18, 20, 21]. Morphological changes in the *mdx* diaphragm progress earlier and more severely compared to the TA [8, 21]. In the diaphragm of *mdx* mice, we see atrophy that is not evident in the TA, earlier onset of fibrotic deposition and no myofibre hypertrophy. Conversely, hypertrophic *mdx* myofibres are maintained in the TA [9, 18]. In *mdx* mice, increases in myofibres and muscle mass precede severe fibrosis and atrophy, which are then followed by concurrent reductions in intramuscular fat and body weight [8, 22]. Compared to DMD patients, *mdx* mice experience delayed fibrosis, reduced intramuscular fat accumulation and generally milder disease progression. Notably, intramuscular fat and muscle strength are inversely proportional in human patients, whereas mice are considerably leaner with more glycolytic myofibres [22, 23]. As previously suggested, metabolism may play a key role in the differing progression of muscular dystrophy across species [8, 22, 24].

Myofibre branching, suspected to originate from aberrant muscle cell fusion during regeneration, contributes to increased myofibre numbers and decreased force production in *mdx* muscle [25, 26, 27, 28, 29]. However, we find that increases in small myofibres and elevated TA mass correlate with the preservation of maximum tetanic force beyond 20 weeks. Normalized force also does not decrease with age, which we would predict if the increase in muscle mass were attributed to pathogenic branching. We thus theorize that the increase in small myofibres results from a combination of myofibre branching and beneficial hyperplasia resulting from ongoing muscle regeneration.

Advances in genomics have improved our understanding of DMD, highlighting the regenerative nature of dystrophin‐deficient muscle. RNA‐seq of whole *mdx* muscle identified upregulated genes related to regenerating muscle and pathway enrichment relating to inflammation, fibrosis, proliferation, necrosis and apoptosis [30]. Single‐nucleus RNA‐seq of the dystrophin‐deficient TA muscle observed changes in the cellular milieu and identified a regenerative myonuclear population [17]. Our transcriptomic analysis provides further evidence of ongoing muscle regeneration by *mdx* MuSCs. We see the full trajectory of myogenic cells in *mdx* muscle and the upregulation of proliferation and differentiation markers in MuSCs. Our study of acutely CTX‐challenged muscle provides further insight into muscle regeneration in the *mdx* mouse and agrees with previous findings [31, 32].

In WT mice, branching occurs in about half of regenerated myofibres following CTX injury [25]. Despite this, maximum titanic and specific force recovers in the WT TA by 21 dpi. However, maximum tetanic force does not recover in the *mdx* TA until 90 dpi. No significant collagen deposition following CTX injury occurs in either genotype; thus, fibrosis likely does not contribute to decreased force production in the *mdx* TA. The decrease in force production may be attributed to increased instances of myofibre branching in *mdx* regenerating muscle; however, myofibre branching was not assessed.

Increased TA weight between 21 and 90 dpi aligns with the finding that 5% of MuSCs are cycling a month after a CTX injury [33]. However, the morphometric myofibre changes following acute injury differ between genotypes. We observe muscle and myofibre hypertrophy in the WT TA and myofibre hyperplasia in the *mdx* TA. This is reminiscent of neonatal myogenesis when young *mdx* myofibres are hypotrophic and contain fewer myonuclei compared to WT controls until 3–4 weeks [9, 17, 18]. Considering our previous findings [2, 5], hypotrophic *mdx* myofibres are an in vivo indicator of deficient muscle regeneration in the *mdx* mouse.

In line with reports in *mdx* mice and DMD patients, we observe an expansion of PAX7^+^ cells in homeostatic conditions and following transplantation [1, 12, 13]. Our findings suggest this results from two mechanisms. First, we histologically and transcriptomically demonstrate that the continual activation of MuSCs by the dystrophic microenvironment leads to elevated numbers of PAX7^+^ progenitors. We also see the upregulation of immune and activation‐related genes in *mdx* myogenic cells, corroborating the previous findings that MuSCs respond to the extrinsic dystrophin microenvironment [34]. Second, our in vivo study of neonatal and CTX‐challenged MuSCs combined with our engraftment data indicate that intrinsic polarity deficits cause *mdx* MuSCs to favour symmetric expansion over asymmetric cell division following activation [2, 5]. Specifically, our scRNA‐seq analysis identifies hyperplasia of uncommitted *mdx* MuSCs and fewer progenitor cells following CTX injury. Additionally, *mdx* MuSCs have impaired PARD3 polarity and generate fewer progenitor cells after CTX injury and in pre‐necrotic neonatal muscle. Finally, despite the similar engraftment of WT and *mdx* MuSCs, we also find that *mdx* MuSCs have significantly reduced regenerative potential, evidenced by smaller grafts and reduced numbers of myofibres, even in the absence of the dystrophic microenvironment. The enhanced self‐renewal capacity of *mdx* MuSCs may result in a competitive growth advantage over WT MuSCs. Consequently, dystrophin‐deficient MuSCs that have undergone gene correction to restore polarity may eventually be lost to a competitive self‐renewal disadvantage.

MuSCs are highly regulated by their niche [S20], and chronic exposure to the dystrophic microenvironment contributes to MuSC dysfunction [34]. For instance, chronic inflammatory cytokine exposure leads to elevated NF‐κB in MuSCs, resulting in telomere shortening after repeat muscle injury [S21]. Moreover, directly antagonizing the inflammatory cytokine IL6 or STAT3 leads to improved dystrophic MuSC differentiation [S22, S23]. Therefore, both extrinsic and intrinsic factors contribute to MuSC dysfunction in muscular dystrophy.

In line with these observations, we find that the total number of MuSCs decreases linearly in *mdx* muscles with age. Chronic inflammation induces cell senescence in dystrophic muscle. However, in young dystrophic mice, senescent cells are predominantly macrophages and endothelial cells, not PAX7^+^ MuSCs [S24]. Thus, the decreased number of *mdx* MuSCs could result from MuSCs become increasingly dysfunctional by the accumulation of non‐myogenic senescent cells. However, the number of senescent MuSCs increases with age in WT mice and in other muscle wasting diseases [S25]. It is also possible that the age‐related decline in *mdx* MuSCs results from MuSC senescence, which may worsen skeletal muscle pathology. Nonetheless, removal of senescent cells with senolytics in ageing mice improves acute injury outcomes [S26]. Another possible contributor to the decline in MuSCs is that dystrophic MuSCs undergo another mechanism of non‐apoptotic arrest through mitotic catastrophe [2].

The current study provides strong evidence that DMD skeletal muscle pathology and MuSC dysfunction are not only attributed to the inflammatory and degenerative mechanisms that result from myofibre fragility. In addition, the absence of dystrophin in MuSCs creates an intrinsic polarity deficit that impairs MuSC asymmetric cell division and commitment, resulting in perturbed muscle regeneration in vivo, as demonstrated by our neonatal and engraftment experiments. Interestingly, studies suggest that dystrophin deficiency in MuSCs and myofibres synergistically drive disease progression. Chronic MuSC activation and myogenesis exacerbate sarcolemma instability and thus myofibre fragility [35, 36, 37], whereas the fusion of *mdx* myocytes is imperative for the integrity of dystrophic muscle [38]. Thus, an attractive therapeutic approach in DMD is targeting mechanisms that restore endogenous MuSC asymmetric division through DGC‐independent mechanisms [39]. This provides a more nuanced approach compared to mechanisms that induce MuSC activation, proliferation and differentiation. Overall, our study underscores the importance of studying and targeting both myofibres and MuSCs in the context of treating DMD.

## Ethics Statement

The authors certify that the manuscript complies with the ethical guidelines for authorship and publishing in the *Journal of Cachexia, Sarcopenia and Muscle* [40]. The manuscript does not contain clinical studies or patient data.

## Conflicts of Interest

M.A.R. is the Founding Scientist and Chief Development Officer of Satellos Bioscience Inc. The other authors declare no conflicts of interest.

## Supporting information


**Table S1** List of antibodies.
**Table S2.** Other reagents.
**Figure S1 (related to Figure 1).** Progressive changes in the *tibialis anterior* occur with age in the *mdx* mouse.
**Figure S2 (related to Figure 2).** Severe pathology occurs in the *mdx* diaphragm.
**Figure S3 (related to Figure 3).** Altered regeneration of the *mdx* muscle.
**Figure S4 (related to Figure 3).** Disrupted regeneration after triple cardiotoxin injury of the *mdx* tibialis anterior.
**Figure S5 (related to Figures 5 and 6).** Bulk RNA‐sequencing identifies transcriptomic differences in wild‐type and *mdx* myogenic cells.
**Figure S6 (related to Figures 5 and 6)**. Sequencing of single myogenic cells identifies cell identity.
**Figure S7.** Impaired *mdx* MuSC polarity results in reduced generation of myocytes.
**Figure 8 (related to Figure 8).** Impaired muscle regeneration by *mdx* MuSCs.

**Data S1.** Supporting Information.

**Data S2.** Supporting Information.

**Data S3.** Supporting Information.
