# Supplementary material for: Intrinsic Muscle Stem Cell Dysfunction Contributes to Impaired Regeneration in the mdx Mouse
Source: J Cachexia Sarcopenia Muscle. 2024 Dec 26;16(1):e13682. doi: 10.1002/jcsm.13682 (PMC11669949; doi:10.1002/jcsm.13682)
Supplement: Supplementary file 1 — Table S1 List of antibodies. Table S2. Other reagents. Figure S1 (related to Figure 1). Progressive changes in the tibialis anterior occur with age in the mdx mouse. Figure S2 (related to Figure 2). Severe pathology occurs in the mdx diaphragm. Figure S3 (related to Figure 3). Altered regeneration of the mdx muscle. Figure S4 (related to Figure 3). Disrupted regeneration after triple cardiotoxin injury of the mdx tibialis anterior. Figure S5 (related to Figures 5 and 6). Bulk RNA‐sequencing identifies transcriptomic differences in wild‐type and mdx myogenic cells. Figure S6 (related to Figures 5 and 6). Sequencing of single myogenic cells identifies cell identity. Figure S7. Impaired mdx MuSC polarity results in reduced generation of myocytes. Figure 8 (related to Figure 8). Impaired muscle regeneration by mdx MuSCs. [file JCSM-16-e13682-s001.pdf]

Supplemental Information for:

Intrinsic muscle stem cell dysfunction contributes to impaired regeneration in the mdx mouse

Marie E. Esper, Caroline E. Brun, Alexander Y. T. Lin, Peter Feige, Marie J. Catenacci, Marie-Claude Sincennes, Morten Ritso, and Michael A. Rudnicki

1. Supplemental Methods
2. Supplemental Acknowledgements
3. Supplemental References
4. Supplemental Figures S1-S8
5. Supplemental Tables S1 and S2

Supplemental methods

Animal models, tamoxifen injections and muscle injury

All experiments were performed on male mice and initiated at 6-10 weeks old unless stated otherwise. The following mouse lines were used: C57BL/10ScSnJ (referred as WT, JAX #000476) and C57BL/10ScSn-Dmd^{mdx}/J (referred as *mdx*, JAX #001801). To facilitate cell sorting, engraftment and sequencing experiments, WT and *mdx* mice were crossed with the following transgenic strains: *CAG-GFP* (JAX #006567), *Pax7nGFP* (Sambasivan, Gayraud-Morel et al. 2009), *ROSA-nTnG* (JAX #023035), *Myf5*^{Cre};*R*^{nTnG} (JAX #007845 and JAX #023035) and *Myf5*^{CreER}; *R26R*^{mTmG} (JAX #023342 and JAX #007576). For muscle injury, mice were anesthetized using isoflurane (2% in oxygen), then injected with 50 µL of 10 µM Cardiotoxin (CTX) solution (Latoxan) directly into the *tibialis anterior* (TA) muscle. For fluorescence cell sorting of activated MuSCs, both TA and *gastrocnemius* muscles were injected with 40 µL and 80 µL of 10 µM CTX, respectively. Mice were provided either 0.1mg/kg buprenorphine (CTX-injury experiments) or 50 µL of 5 mg/mL carprofen (polarity and engraftment experiments) for pain management. To obtain neonatal mice, breeding pairs were monitored for births after 18 days in the same enclosure. The day of discovery was labelled as perinatal day 0 (P0).

In situ force measurement

Mice were anesthetized using isoflurane (2% in oxygen) by nose cone throughout the procedure. Muscle force measurements were performed on TA muscle using an Aurora Scientific 300C-LR-FP dual mode muscle lever system equipped with a 1N force transducer and 1cm lever arm, as previously described[1]. Electrical stimulation was performed using monopolar needle electrodes attached to an Aurora Scientific 701C High-Power, Bi-Phase Stimulator. Force transducers were calibrated prior to each study using precision weights.

Two monopolar needle electrodes were positioned adjacent to the tibial nerve proximal to the kneecap and distal to the kneecap adjacent the extensor digitorum longus muscle. Traction was applied to the transducer to maintain 20 mN of measured tension for an initial 15 min stretching period with 100 ms

trains of 0.3 ms, 5 V supramaximal voltage pulses at 1 Hz stimulation every 100 seconds. Following stretching, muscles were maintained at 20 mN static tension and tetanic contractions were measured every 100 seconds following 200 ms trains of 0.3 ms, 5V supramaximal voltage pulses at serial frequencies from 1 Hz to 200 Hz. Maximal force was defined by the difference in maximal force measured during stimulation to that of the static tension immediately prior to stimulation.

Following force measurement, muscle mass and volume were measured. Specific force of muscles was calculated as follows: $\frac{F_{measured}}{PCSA}$ and $PSCA = \frac{M_{displacement}}{M_{FL}} \times Cos(M_{PA})$, where $F_{measured}$ is the force measured in mN, $PSCA$ is the physiological cross-sectional area in mm^2, $M_{displacement}$ is the measured muscle displacement in µL (mm^3), M_{FL} is the experimentally determined muscle fiber length in mm, M_{PA} is the experimentally determined muscle pennation angle in °. For non-volumetric analysis, muscle mass was related to the assumed density of mammalian tissue (1.06g.m^{-3}). Muscle fiber length and pennation angles were determined previously.[2]

Section staining for histology

In the aging and acute injury studies, mice were euthanized, and TA muscles were harvested, weighed, and embedded in Tissue-Tek OCT and frozen in liquid nitrogen-cooled isopentane. Embedded muscles were transversely sectioned at 10 µm-thickness.

Masson's trichrome staining. Muscle sections were fixed in Bouin's solution overnight. After washing in tap water, sections were incubated in Weigert's hematoxylin solution for 10 min and rinsed under running tap water for 10 min. After washing in distilled water, sections were stained with Biebrich scarlet-acid fuchsin solution for 5 min and washed again in distilled water. Sections were then incubated in a phosphomolybdic-phosphotungstic acid solution for 15 min and transferred in aniline blue solution for 5 min. Sections were briefly rinsed in water and incubated in 1% glacial acetic acid for 2 min. After washing in distilled water, sections were dehydrated in a 95-100% graded ethanol series and cleared in xylene for 5min. Samples were mounted using DPX mounting media.

Immunostaining. Cross-sections were fixed in 4% PFA 10 min at room temperature, permeabilized in 0.1M glycine, 0.1% Triton X-100 in PBS, and blocked in 5% goat serum, 2% BSA in PBS supplemented with M.O.M. Blocking reagent (Vector Laboratories). Sections were then incubated with primary antibodies overnight at 4°C. The list of primary antibodies is available in **Table S1**. Samples were washed 3 times in PBS, incubated 1h at room temperature with Alexa Fluor-conjugated cross absorbed secondary antibodies at 1:1,000 (ThermoFisher), washed 3 times in PBS and counterstained with Hoechst or DAPI at 1µg/mL in PBS before mounting.

Histological analysis

Cross-sectional area. Acute and chronic injury images were taken on a Zeiss Axio Observer.D1 inverted microscope or a Zeiss Axio Imager M2 Microscope equipped with a 10x objective and stitched using Fiji software (http://fiji.sc/Fiji). Trichrome-stained cross-sections were used to measure total cross-sectional area using the trace tool in Fiji. Transplantation images were acquired using a Zeiss Axio Observer.D1 and stitching using Fiji (2 weeks) or a Zeiss Observer7 using a 20x apo objective and stitched using Zeiss ZEN 3.7 blue edition software (4 weeks).

Minimum Feret's diameter. Laminin immunostaining was used to delineate myofibers. For the acute and chronic injury experiments, Minimum Feret's diameters were measured using the semi-automated SMASH software plugin for MATLAB 2015a.[3] Over 90% of myofibers were quantified for each cross-section studied. FIJI's analyze particle function was used to assess Minimum Feret's diameters for the transplantation study.

Fiber number. Total myofiber count per cross-section was verified by manual validation of SMASH myofiber masks and original images of injury series experiments for acute and chronic injury time course. For transplantation studies, the total myofiber count per cross-section were enumerated using FIJI's analyze particle function.

Collagen deposits. Collagen area was analyzed using Masson's trichrome staining by measuring the area of thresholded pixels in images extracted from the red channel. The positive stained tissue was calculated as a fraction of the total cross-sectional area.

Lipid droplets. Fat infiltration was calculated using Bodipy staining by measuring the area of thresholded pixels in Bodipy single channel images. The positive stained tissue was calculated as a fraction of the cross-sectional area.

Diaphragm thickness measurements. Diaphragm hypertrophy was assessed by measuring the thickness of diaphragm cross-sections at a minimum of 15 points along each muscle using FIJI.

Muscle stem cell number. Chronic and acute injury images of PAX7 immunostaining were taken on a Zeiss Axio Observer.D1 inverted microscope using a 20x apochromatic objective and analyzed on ZEN software. The number of PAX7^{+} cells per mm^{2} was averaged from 6 to 12 fields to cover more than 90% of the tissue. During disease progression, we aimed to compare regions of non-acute injury, thus abnormal frames with exceptionally high numbers of PAX7 cells and small centrally nucleated myofibers (regions of micro injury) were excluded. For transplantation study and neonatal sections, the entire graft/muscle was imaged using a Zeiss Axio Observer.D1 and stitching using Fiji (2 week grafts) or a Zeiss Observer7 (4 week grafts and neonatal muscle) using a 20x apochromatic objective and stitched using Zeiss ZEN 3.7 blue edition software. The entire graft was enumerated for PAX7^{+} cells, while the entire *tibialis anterior* (TA) and *extensor digitorum longus* (EDL) cross-section was enumerated for neonatal hindlimb sections.

Fluorescence-activated cell sorting (FACS)

MuSCs isolated from hindlimb muscles were dissected, minced to 1mm sized pieces, and dissociated in collagenase/dispase solution using a gentle MACS Octo Dissociator (Miltenyi Biotec 130-095-937). The slurry was filtered through a 100µm filter, pelleted, and treated with red blood cell lysis buffer (Sigma, Hybri-Max, R7757). Staining was performed in 1mL volume using lineage-negative (Lin^{-}) antibodies against CD31, CD11b, CD45, and SCA1 conjugated to BV421, viability dye (7-AAD) and positive selection markers specified under each experimental method. Antibodies are listed in **Table S1**.

Single cell polarity assay

To assess MuSC polarity, Lin^{-} α7-INTEGRIN^{+} VCAM1^{+} cells were FACS-isolated from CTX injured hindlimb muscle 48 hours following injury or from P7 *Pax7-nGFP* limb muscles. Following a short

dissociation (30 min for adult MuSCs; 20 min for neonatal MuSCs) and cell staining (adult MuSCs only), an additional 10-minute fixation with 4% paraformaldehyde was conducted prior to cell sorting to preserve the polarization of polarity proteins. The cells were then adhered to 8-well ibidi slides coated with Cell-Tak (Corning™), permeabilized and stained with an antibody for PARD3 (1:500, Millipore - 07330). Adult MuSC images were acquired with a Zeiss Axio Observer.D1equipped with a 63x oil objective and analysed using Zen Software. Neonatal MuSC images were acquired using an LSM900 using a 20x apochromatic objective and stitched using Zeiss ZEN 3.7. Polarization was identified by PARD3 partitioning in no more than half of the cell's cytoplasm. Greater than 500-1000 adult MuSCs were quantified per condition, and an average of 536 neonatal MuSCs were examined per replicate.

Satellite cell engraftment assay

Transplantation experiments were modified from previously described protocols.[4] Recipient immunodeficient NSG (NOD.Cg-*Prkdc*^{scid} *Il2rg*^{tm1Wjl}*/SzJ*, JAX #005557) mice were anesthetized by isofluorane and treated with subcutaneous carprofen (50 µL of 5 mg/ml). Under anesthesia, the hindlimbs were irradiated with 8Gy X-rays delivered at 0.71 Gy.min^{-1} (X-RAD 320, Precision X-Ray) using a lead barrier to protect the body. Immediately following irradiation, the TA was injured using 50µL of 10µM Cardiotoxin (Latoxan). Two days later, 10,000 donor MuSCs from 6-8-week-old WT and *mdx CAG-GFP* or *ROSA-nTnG* mice were injected into the contralateral TAs of NSG mice. Fluorescent donor MuSC (tdTomato^{+} or GFP^{+}) were FACS-isolated based on FSC/SSC, viability (7-AAD^{-}), lineage negative selection (CD31^{-}, CD11b^{-}, CD45^{-}, SCA1^{-}), and positive selection (α7-INTEGRIN^{+}, VCAM1^{+}). Donor muscle stem cells were washed with PBS and resuspended in 0.9% NaCl solution at a concentration of 10,000 cells per 20µL prior to engraftment into the TA muscle of irradiated and injured NSG mice. Two- and four-weeks following transplantation, NSG mice were perfusion-fixed and processed as previously described.[5] Briefly, the fixed TA muscles were harvested, fixed overnight in 1% PFA at 4°C, submerged in a 15% to 30% sucrose gradient at 4°C, embedded in Tissue-Tek OCT and frozen in liquid nitrogen-cooled isopentane. Embedded muscles were transversely sectioned at 10 µm-thickness. Antibody staining was

performed as described above beginning from the permeabilization step. Antibodies are listed in **Table S1**.

Bulk RNA-sequencing library generation and gene expression analysis

Myoblast isolation, culture, and RNA extraction. Primary myoblasts were derived from hindlimb muscles of 6–8-week-old male mice by magnetic cell separation (MACS) as previously described.[6] Briefly, cells were isolated based on lineage-negative markers ($CD31^-$, $CD11b^-$, $CD45^-$, $SCA1^-$) and positive selection for α7-INTEGRIN. Myoblasts were cultured on collagen-coated dishes in Ham's F10 medium (Wisent, 318-051-CL) supplemented with 20% FBS, 1% penicillin/streptomycin, and 5ng/mL of basic FGF (Millipore, GF003AFMG). Myoblast differentiation was induced for 2 days in Ham's F10:DMEM 1:1 (Wisent, 319-016-CL) supplemented with 5% horse serum, and 1% penicillin/streptomycin. Total RNA was extracted using the Nucleospin RNA II kit (Macherey-Nagel), according to the manufacturer's instructions.

MuSC isolation and RNA extraction. MuSCs were obtained from uninjured and 3-day CTX-injured *Pax7-nGFP* TA and *gastrocnemius* muscle. MuSCs were sorted by gating a mononuclear cell population of α7-$INTEGRIN^+$, GFP^+ ($PAX7^+$), $CD31^-$, $CD45^-$, $SCA1^-$, $CD11b^-$ using a MoFlo XDP cell sorter (Beckman Coulter). Antibodies are listed in **Table S1**. Total RNA was isolated using Arcturus Picopure RNA extraction kit (Thermo Fisher).

Sample sizes. 24 biological samples were used for RNA-sequencing analysis: n = 3 quiescent MuSCs, n = 3 activated MuSCs, n = 3 myoblasts and n = 3 myotubes, for each genotype (WT and *mdx*). For quiescent MuSCs, all hindlimb muscles from 2-3 mice were combined to obtain one sample. For activated MuSCs, myoblasts and myotubes, each sample corresponded to one mouse.

Library construction, sequencing, and analysis. Library construction was performed with 20ng of input total RNA using the NEBNext Ultra II Directional RNA Library Prep Kit for Illumina - polyA mRNA workflow (New England Biolabs). The libraries were sequenced with a NextSeq 500 High Output 75 cycle kit (Illumina). RNA-seq reads were mapped to transcripts from GRCm38_GENCODE.vM19 using salmon v0.13.1.[7] The data was loaded into R using the tximport package and the gene/count matrix was

filtered to retain only genes with five or more mapped reads in two or more samples. Differential gene expression testing and PCA plotting was performed using DESeq2.[8] An adjusted *p*-value less than 0.05 defined differentially expressed genes. Fold change was determined using lfcShrink, applying the apeglm method (v1.6.0).[9] Differentially expressed genes were based on a log2 fold change greater than 2 and an adjusted *p*-value less than 0.05. Functional analysis of the differentially expressed genes were examined using Gene Ontology Biological Processes,[10,11] and the R application clusterProfiler.[12]

Single-cell RNA-sequencing and analysis

WT and *mdx Myf5creER;ROSA-mTmG* were subjected to four intraperitoneal tamoxifen injections (100 μL of 20 mg/mL in corn oil) at 24h intervals prior to injury. MuSCs (mGFP$^+$ α7-INTEGRIN$^+$,GFP$^+$, CD31$^-$, CD45$^-$, SCA1$^-$, CD11b$^-$) were isolated from the uninjured and 5-days post CTX injured hindlimb muscles of male WT and *mdx* tamoxifen-treated *Myf5creER;ROSA-mTmG* mice. Antibodies are listed in **Table S1**. The isolated cells were then captured using the 10X Genomics Chromium Platform and sequenced to a mean read depth of 21,000 reads/cell using the Illumina NextSeq 500. The CellRanger v7.1.0 pipeline (10X Genomics) with default parameters was used to align reads to GENCODE vM25 (GRCm38/mm10).

MuSCs were isolated from the uninjured and 5-day CTX injured hindlimb muscles of male WT and *mdx* tamoxifen-treated *Myf5creER;ROSA-mTmG* mice. To capture the entire trajectory of mononuclear myogenic cells, mGFP$^+$ α7-INTEGRIN$^+$,GFP$^+$, CD31$^-$, CD45$^-$, SCA1$^-$, CD11b$^-$ cells were isolated from the hindlimb muscle of male WT and *mdx* tamoxifen-treated *Myf5creER;ROSA-mTmG* mice by FACS. WT and *mdx Myf5creER;ROSA-mTmG* were subjected to four intraperitoneal tamoxifen injections (100 μL of 20 mg/mL in corn oil) at 24h intervals prior to injury and cell isolation. Antibodies are listed in **Table S1**. The isolated cells were then captured using the 10X Genomics Chromium Platform and sequenced to a mean read depth of 21,000 reads/cell using the Illumina NextSeq 500. The CellRanger v7.1.0 pipeline (10X Genomics) with default parameters was used to align reads to GENCODE vM25 (GRCm38/mm10).

Filtering, qualify control and analysis was performed using R (v4.3.1) and the package Seurat (v4.3.0).[13] Genes expressed in less than 3 cells and cells expressing fewer than 200 genes were excluded from the analysis. Cells with more than 30% mitochondrial reads were also removed. Following cell doublet removal using scDblFinder,[14] a total of 4205 WT and 6980 *mdx* high quality cells were captured in the uninjured libraries. Similarly, 4616 WT and 5396 *mdx* cells were captured in the injured libraries.

Individual libraries were analysed separately prior to integration using standard Seurat processing steps. We then performed Harmony integration on the combined data using the RNA assay.[15] Following normalization and scaling of the data, we performed principal component analysis on the top 3000 genes in the integrated object, followed by Uniform Manifold Approximation Projection (UMAP) embedding using the top 20 principal components. Clustering was performed using a resolution of 0.3, after which cluster were annotated using a combination of known cell markers and Seurat's Find All Markers function that were inputted into the online tool Metascape.[16] The AddModuleScore Seurat function was used to evaluate the enrichment of 400 quiescence-enriched genes.[17] Publication graphics were generated using Seurat, SCpubr and scCustomize.[13,18,19]

Statistical analysis

Statistical evaluation was performed using the Student's two tailed *t*-test tests to calculate differences between two groups and either one-way or two-way ANOVA with post hoc test for multiple comparisons (Graphpad Prism 10®). The number of independent experimental replications is reported in each corresponding figure legend. Unless otherwise stated, data are presented as mean ± SEM and p-value < 0.05 was considered as statistically significant. Level of significance is indicated as follows: $*p < 0.05$, $**p < 0.01$, $***p < 0.001$.

Supplemental acknowledgements

The authors thank Jennifer Ritchie, GuoHua Li, and Dallas Bennett for animal husbandry, Fernando Ortiz for FACS and flow assistance, Christopher Porter for bioinformatics support, Sandy Martino for administrative assistance, and Dr. Derek Hall for the helpful discussions while preparing this manuscript. M.E.E. was supported by the Queen Elizabeth II Graduate Scholarship (QEII-GSST) and the Ontario Graduate Scholarship (OGS). C.E.B. was supported by postdoctoral fellowships from the Ontario Institute for Regenerative Medicine (OIRM), the French Muscular Dystrophy Association (AFM)-Téléthon and the Fondation pour la Recherche Médicale (FRM, ARF201909009155). P.F. was supported by a doctoral fellowship from the Canadian Institutes of Health Research (CIHR). A.Y.T.L. was supported by a postdoctoral fellowship from the OIRM. M.R. was supported by the University of Ottawa Brain and Mind Research Institute's CNMD STaR award.

Supplemental References

1. Brun CE, Sincennes M-C, Lin AYT, Hall D, Jarassier W, Feige P *et al.* GLI3 regulates muscle stem cell entry into GAlert and self-renewal. *Nat Commun* 2022;**13**:3961.

2. Charles JP, Cappellari O, Spence AJ, Hutchinson JR, Wells DJ. Musculoskeletal Geometry, Muscle Architecture and Functional Specialisations of the Mouse Hindlimb. *PLoS One* 2016;**11**:e0147669.

3. Smith LR, Barton ER. SMASH – semi-automatic muscle analysis using segmentation of histology: a MATLAB application. *Skeletal Muscle* 2014;**4**:1–16.

4. Feige P, Rudnicki MA. Isolation of satellite cells and transplantation into mice for lineage tracing in muscle. *Nat Protoc* 2020;**15**:1082–1097.

5. Esper ME, Kodippili K, Rudnicki MA. Immunofluorescence Labeling of Skeletal Muscle in Development, Regeneration, and Disease. *Methods Mol Biol* 2023;**2566**:113–132.

6. Sincennes MC, Wang YX, Rudnicki MA. Primary Mouse Myoblast Purification using Magnetic Cell Separation. In: Perdiguero E, Cornelison D, editors. *Muscle Stem Cells: Methods and Protocols*. Springer: New York, NY; 2017. pp. 41–50.

7. Patro R, Duggal G, Love MI, Irizarry RA, Kingsford C. Salmon provides fast and bias-aware quantification of transcript expression. *Nature Methods* 2017;**14**:417–419.

8. Love MI, Huber W, Anders S. Moderated estimation of fold change and dispersion for RNA-seq data with DESeq2. *Genome Biol* 2014;**15**:550.

9. Zhu A, Ibrahim JG, Love MI. Heavy-tailed prior distributions for sequence count data: removing the noise and preserving large differences. *Bioinformatics* 2019;**35**:2084–2092.

10. Ashburner M, Ball CA, Blake JA, Botstein D, Butler H, Cherry JM *et al.* Gene Ontology: tool for the unification of biology. *Nat Genet* 2000;**25**:25–29.

11. The Gene Ontology Consortium, Aleksander SA, Balhoff J, Carbon S, Cherry JM, Drabkin HJ *et al.* The Gene Ontology knowledgebase in 2023. *Genetics* 2023;**224**:iyad031.

12. Wu T, Hu E, Xu S, Chen M, Guo P, Dai Z *et al.* clusterProfiler 4.0: A universal enrichment tool for interpreting omics data. *The Innovation* 2021;**2**:100141.

13. Hao Y, Hao S, Andersen-Nissen E, Mauck WM, Zheng S, Butler A *et al.* Integrated analysis of multimodal single-cell data. *Cell* 2021;**184**:3573-3587.e29.

14. Germain P-L, Lun A, Garcia Meixide C, Macnair W, Robinson MD. Doublet identification in single-cell sequencing data using scDblFinder. *F1000Res* 2021;**10**:979.

15. Korsunsky I, Millard N, Fan J, Slowikowski K, Zhang F, Wei K *et al.* Fast, sensitive and accurate integration of single-cell data with Harmony. *Nat Methods* 2019;**16**:1289–1296.

16. Zhou Y, Zhou B, Pache L, Chang M, Khodabakhshi AH, Tanaseichuk O *et al.* Metascape provides a biologist-oriented resource for the analysis of systems-level datasets. *Nat Commun* 2019;**10**:1523.

17. García-Prat L, Perdiguero E, Alonso-Martín S, Dell'Orso S, Ravichandran S, Brooks SR *et al.* FoxO maintains a genuine muscle stem-cell quiescent state until geriatric age. *Nat Cell Biol* 2020;**22**:1307–1318.

18. Blanco-Carmona E. Generating publication ready visualizations for Single Cell transcriptomics using SCpubr. 2022;2022.02.28.482303.

19. Marsh S, Salmon M, Hoffman P. scCustomize: Custom Visualizations & Functions for Streamlined Analyses of Single Cell Sequencing. 2024 doi:10.5281/zenodo.10724532.

20. Dinulovic I, Furrer R, Handschin C. Plasticity of the Muscle Stem Cell Microenvironment. *Adv Exp Med Biol* 2017;**1041**:141–169.

21. Tichy ED, Ma N, Sidibe D, Loro E, Kocan J, Chen DZ *et al.* Persistent NF-κB activation in muscle stem cells induces proliferation-independent telomere shortening. *Cell Rep* 2021;**35**:109098.

22. Tierney MT, Aydogdu T, Sala D, Malecova B, Gatto S, Puri PL *et al.* STAT3 signaling controls satellite cell expansion and skeletal muscle repair. *Nat Med* 2014;**20**:1182–1186.

23. Pelosi L, Berardinelli MG, De Pasquale L, Nicoletti C, D'Amico A, Carvello F *et al.* Functional and Morphological Improvement of Dystrophic Muscle by Interleukin 6 Receptor Blockade. *EBioMedicine* 2015;**2**:285–293.

24. Young LV, Morrison W, Campbell C, Moore EC, Arsenault MG, Dial AG *et al.* Loss of dystrophin expression in skeletal muscle is associated with senescence of macrophages and endothelial cells. *Am J Physiol Cell Physiol* 2021;**321**:C94–C103.

25. Saito Y, Chikenji TS. Diverse Roles of Cellular Senescence in Skeletal Muscle Inflammation, Regeneration, and Therapeutics. *Front Pharmacol* 2021;**12**.

26. Moiseeva V, Cisneros A, Sica V, Deryagin O, Lai Y, Jung S *et al.* Senescence atlas reveals an aged-like inflamed niche that blunts muscle regeneration. *Nature* 2023;**613**:169–178.

Supplemental Figures

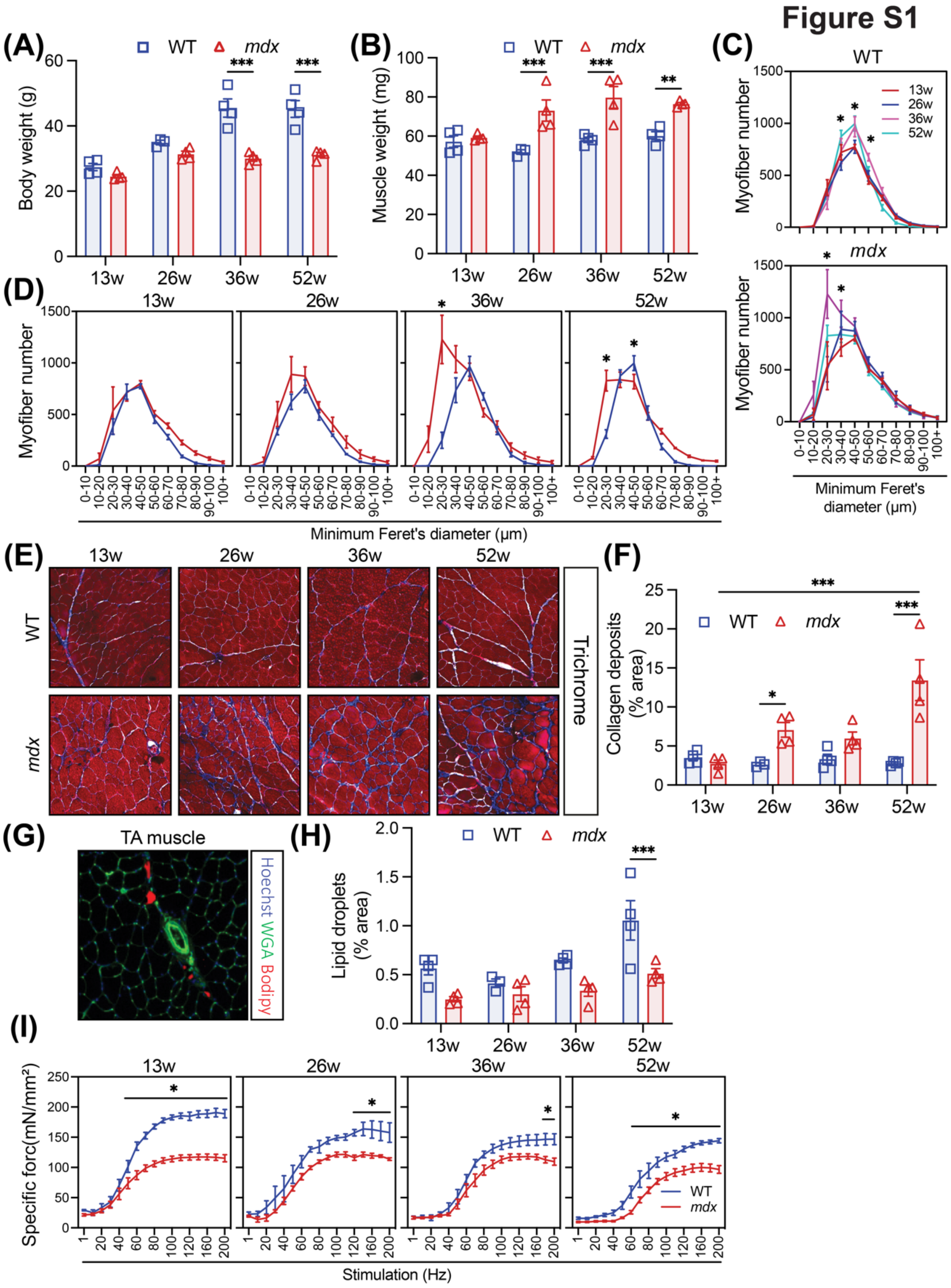

Figure S1 (related to Figure 1). Progressive changes in the *tibialis anterior* occur with age in the *mdx* mouse.

(A) Body weight of wild-type (WT) and *mdx* mice from 13 to 56 weeks of age. (B) Weight of WT and *mdx tibialis anterior* (TA) muscle from 13 to 56 weeks of age. (C) TA myofiber size distribution using minimum Feret's diameter divided by genotype. (D) TA myofiber size distribution using minimum Feret's diameter and divided by age. (E) Representative Trichrome staining of TA cross-sections 13 to 56 weeks of age. (F) Quantification of collagen content (blue) in TA muscle cross-sections stained by Trichrome from13 to 56-week-old WT and *mdx* mice. (G) Representative image of lipid droplets labelled by Bodipy on TA muscle cross-section. (H) Lipid droplet quantification in WT and *mdx* TA cross-sections from 13 to 56 weeks of age. (I) Specific force of WT and *mdx* TA muscle from 13 to 56 weeks of age. Data presented as mean values ± SEM. Two-way ANOVA corrected for multiple comparisons using Sidak's test (*$p < 0.05$; **$p < 0.01$; ***$p < 0.001$).

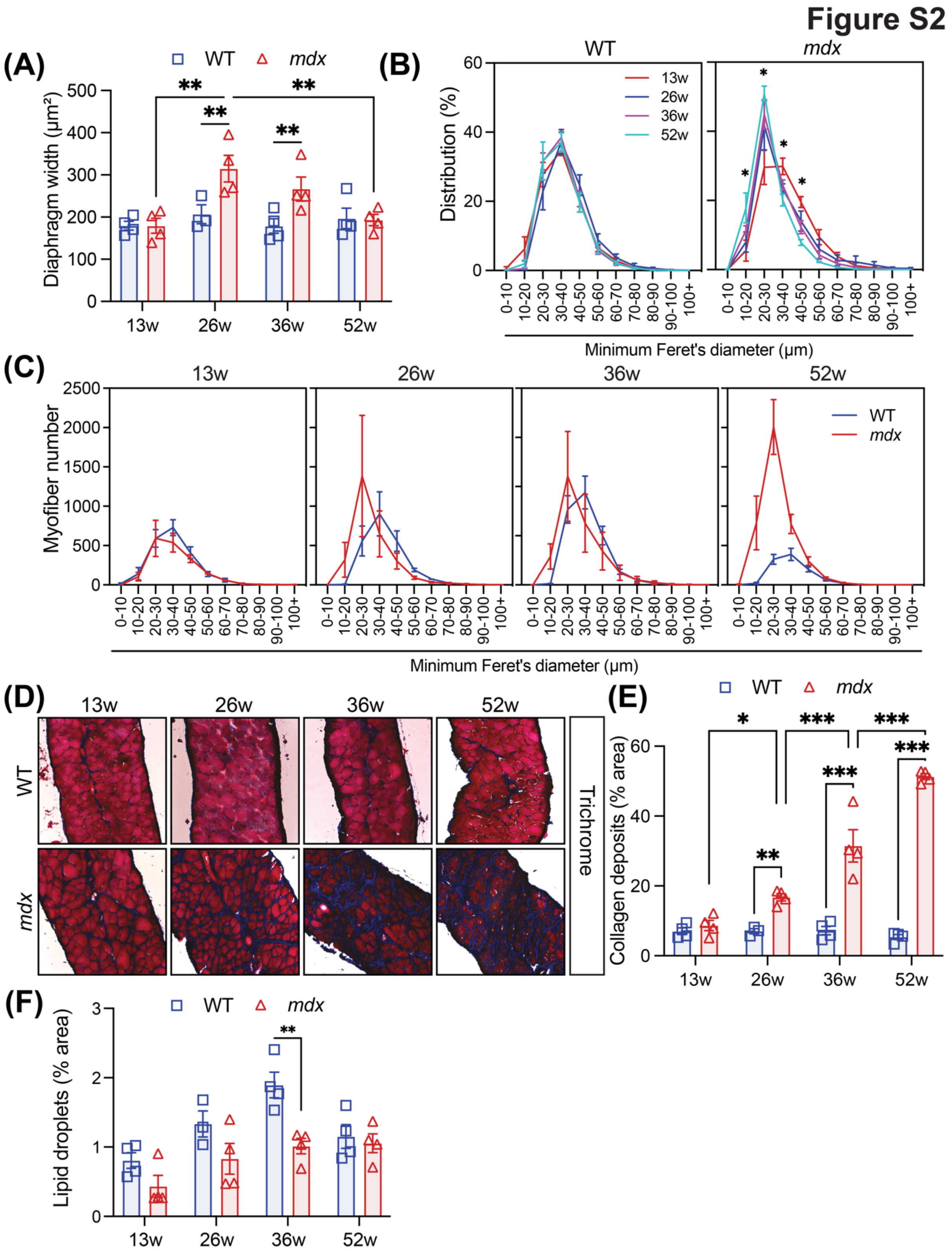
Figure S2
(A)
WT
mdx
Diaphragm width (µm²)
13w
26w
36w
52w
(B)
WT
mdx
Distribution (%)
13w
26w
36w
52w
Minimum Feret's diameter (µm)
(C)
Myofiber number
Minimum Feret's diameter (µm)
(D)
Trichrome
(E)
Collagen deposits (% area)
(F)
Lipid droplets (% area)

Figure S2 (related to Figure 2). Severe pathology occurs in the *mdx* diaphragm.

(A) Wild-type (WT) and *mdx* diaphragm cross-section width from 13 to 56 weeks of age. (B) Normalized diaphragm myofiber size distribution according to size using minimum Feret's diameter and divided by genotype. (C) TA myofiber size distribution using minimum Feret's diameter and divided by age. (D) Representative images of Trichrome staining of diaphragm muscle cross-sections from 13- to 56-week-old mice. (E) Quantification of collagen content (blue) in WT and *mdx* diaphragm muscle sections stained by Trichrome from 13 to 56 weeks. (F) Lipid droplet quantification in diaphragm muscle sections from 13 to 56-week-old WT and *mdx* mice. Data presented as mean values ± SEM. Two-way ANOVA corrected for multiple comparisons using Sidak's test (*$p < 0.05$; **$p < 0.01$; ***$p < 0.001$).

Figure S3

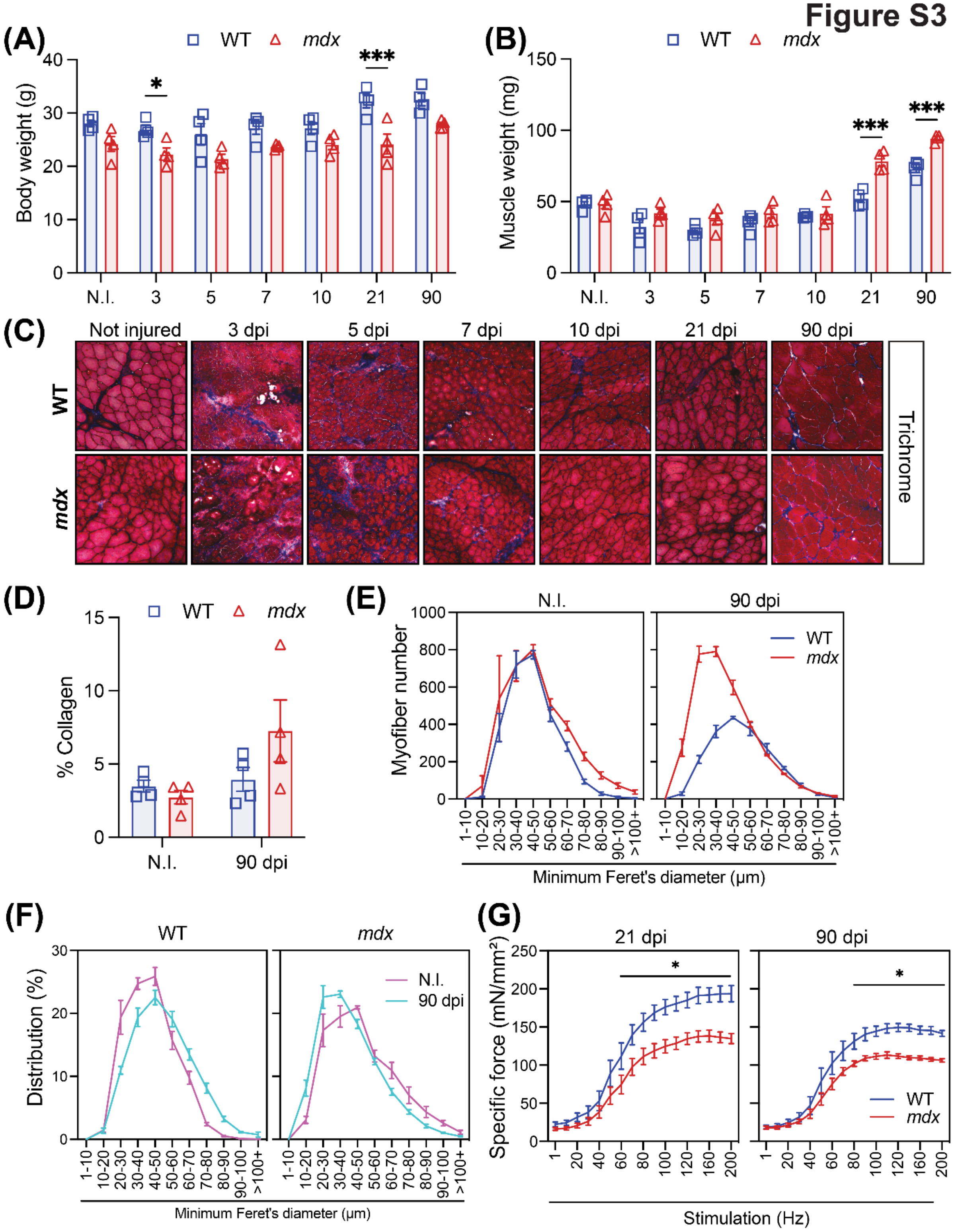
(A)
WT mdx
Body weight (g)
N.I. 3 5 7 10 21 90
(B)
Muscle weight (mg)
(C)
Not injured
3 dpi
5 dpi
7 dpi
10 dpi
21 dpi
90 dpi
WT
mdx
Trichrome
(D)
% Collagen
N.I.
90 dpi
(E)
N.I.
90 dpi
Myofiber number
Minimum Feret's diameter (μm)
(F)
WT
mdx
Distribution (%)
N.I.
90 dpi
Minimum Feret's diameter (μm)
(G)
21 dpi
90 dpi
Specific force (mN/mm²)
Stimulation (Hz)

Figure S3 (related to Figure 3). Altered regeneration of the *mdx* muscle.

A) Body weight of 8- to 13-week-old wild-type (WT) and *mdx* mice before cardiotoxin (CTX)-induced injury (N.I., not injured) until 90 days post-injury (dpi). (B) *Tibialis anterior* (TA) muscle weight of 8- to 13-week WT and *mdx* mice before and following CTX-injury. (C) Representative Trichrome staining images of not injured and injured WT and *mdx* TA muscle cross-sections. (D) Quantification of collagen content (blue staining) in Trichrome-stained TA muscle sections. (E) TA myofiber size distribution using minimum Feret's diameter. (F) Proportional distribution of TA myofiber size divided by genotype.
(G) Specific force of WT and *mdx* TA muscle at 21 and 90 dpi. Data presented as mean values ± SEM. Two-way ANOVA corrected for multiple comparisons using Sidak's test (*$p < 0.05$; **$p < 0.01$; ***$p < 0.001$).

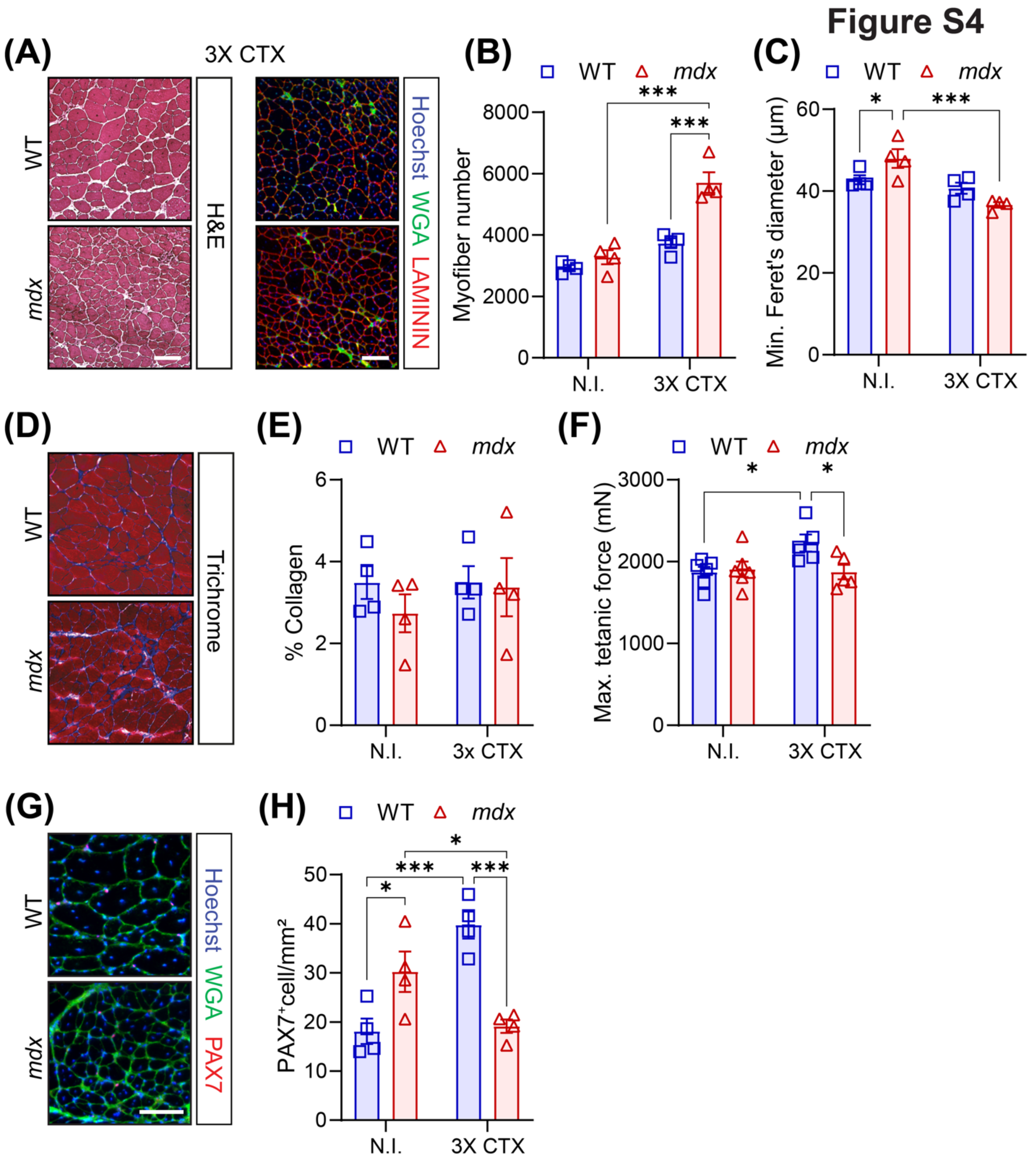

Figure S4 (related to Figure 3). Disrupted regeneration after triple cardiotoxin-injury of the *mdx* tibialis anterior.

(A) Representative hematoxylin and eosin (H&E) and immunofluorescence images of transversal sections of non-injured (N.I.) and triple cardiotoxin (CTX)-injured *tibialis anterior* (TA) muscle from wild-type

(WT) and *mdx* mice. Re-injury conduced every 21 days for a total experiment length of 63 days (3X CTX). LAMININ (red) delineates the myofibers, WGA (green) stains the connective tissues and Hoechst (blue) labels the nuclei. (B) Cross-section TA myofiber quantification after triple CTX-injury compared to N.I. (C) Mean WT and *mdx* TA myofiber size using the minimum Feret's diameter in N.I. and triple CTX-injured muscles (D) Representative Trichrome staining images of N.I. and injured WT and *mdx* TA muscle cross-sections. (E) Quantification of collagen content (blue staining) in Trichrome-stained TA muscle sections. (F) Maximum tetanic force of TA muscle from N.I. and triple CTX-injured WT and *mdx* mice. (G) Representative immunofluorescence images of WT and *mdx* N.I. and triple CTX-injured TA cross-sections from WT and *mdx* mice. PAX7 (red) marks the muscle stem cells (MuSCs), WGA (green) stains the connective tissues and Hoechst (blue) labels the nuclei. (H) PAX7-expressing MuSC number per mm^2 on TA cross-sections. n = 4 mice/genotype. Data presented as mean values ± SEM. Two-way ANOVA corrected for multiple comparisons using Sidak's test (*$p < 0.05$; **$p < 0.01$; ***$p < 0.001$).

Figure S5

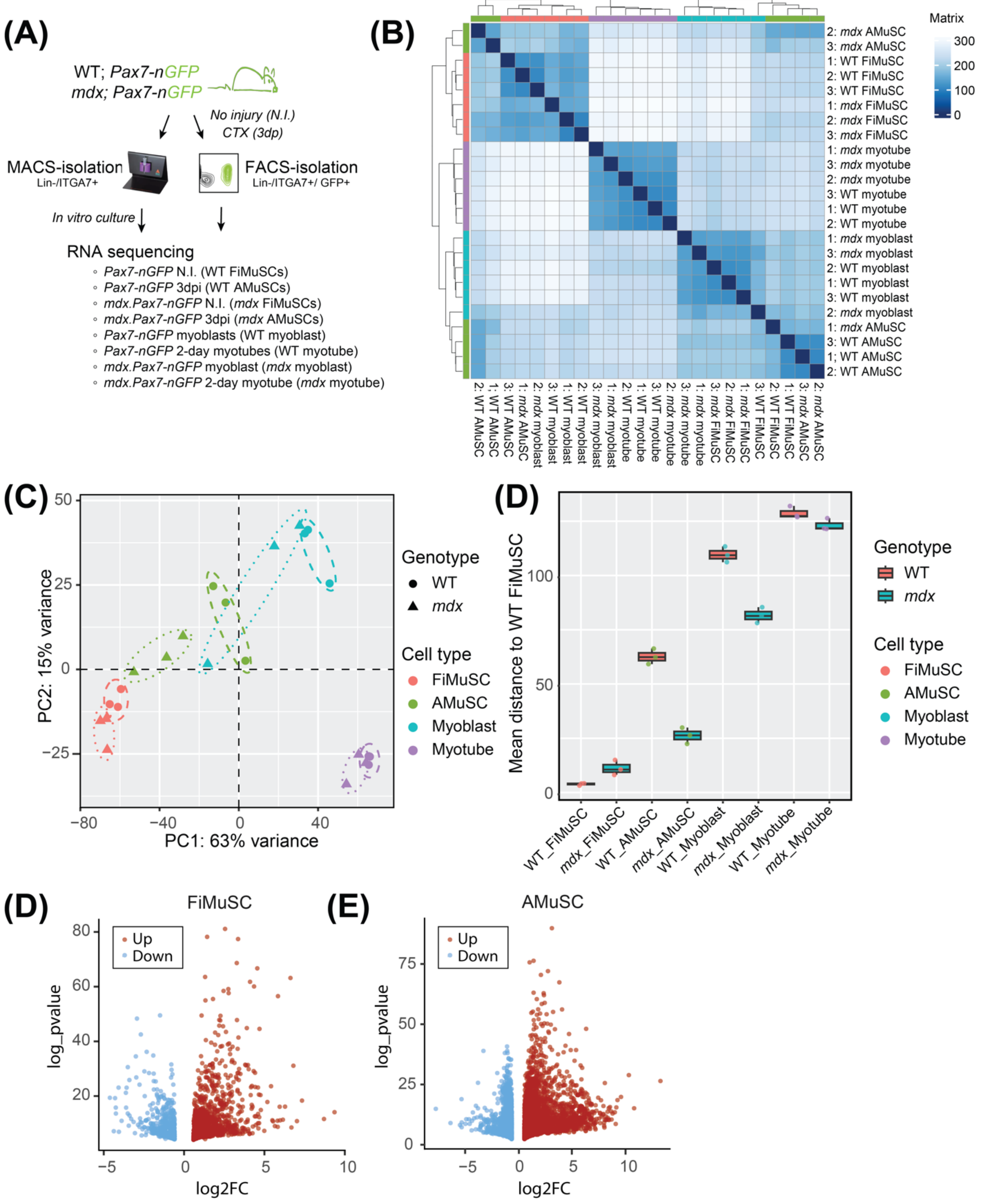
(A)
WT; Pax7-nGFP
mdx; Pax7-nGFP
No injury (N.I.)
CTX (3dp)
MACS-isolation
Lin-/ITGA7+
FACS-isolation
Lin-/ITGA7+/ GFP+
In vitro culture
RNA sequencing
Pax7-nGFP N.I. (WT FiMuSCs)
Pax7-nGFP 3dpi (WT AMuSCs)
mdx.Pax7-nGFP N.I. (mdx FiMuSCs)
mdx.Pax7-nGFP 3dpi (mdx AMuSCs)
Pax7-nGFP myoblasts (WT myoblast)
Pax7-nGFP 2-day myotubes (WT myotube)
mdx.Pax7-nGFP myoblast (mdx myoblast)
mdx.Pax7-nGFP 2-day myotube (mdx myotube)
(B)
Matrix
300
200
100
0
(C)
Genotype
WT
mdx
Cell type
FiMuSC
AMuSC
Myoblast
Myotube
PC1: 63% variance
PC2: 15% variance
(D)
Mean distance to WT FiMuSC
WT_FiMuSC
mdx_FiMuSC
WT_AMuSC
mdx_AMuSC
WT_Myoblast
mdx_Myoblast
WT_Myotube
mdx_Myotube
(D)
FiMuSC
Up
Down
log_pvalue
log2FC
(E)
AMuSC
Up
Down
log_pvalue
log2FC

Figure S5 (related to Figures 5 & 6). Bulk RNA-sequencing identifies transcriptomic differences in wild-type and *mdx* myogenic cells.

(A) Experimental design for fluorescence sorting and sequencing of the total population of GFP^+ $ITGA7^+$ freshly isolated (Fi) and activated (A) muscle stem cells (MuSCs) from the wild-type (WT) and *mdx Pax7-nGFP* mice. *In vitro* myoblasts (MBs) derived from MuSCs of WT and *mdx Pax7-nGFP* mice were amplified or differentiated for 2 days before sequencing. Biological triplicates were included for each genotype and condition (n = 3 biological samples × 4 conditions). (B) Correlation heat map of bulk RNA-sequencing libraries following differential gene expression analysis of *mdx* FiMuSC and AMuSCs libraries compared to WT. (C) Principal component analysis of global transcriptomes of WT and *mdx* FiMuSCs, AMuSCs, myoblasts and 2-day differentiated myotubes. (D) Mean distance on PCA plot to WT FiMuSCs. (E,F) Volcano plot showing the significantly down- and up-regulated genes in *mdx* FiMuSCs and AMuSCs libraries compared to WT.

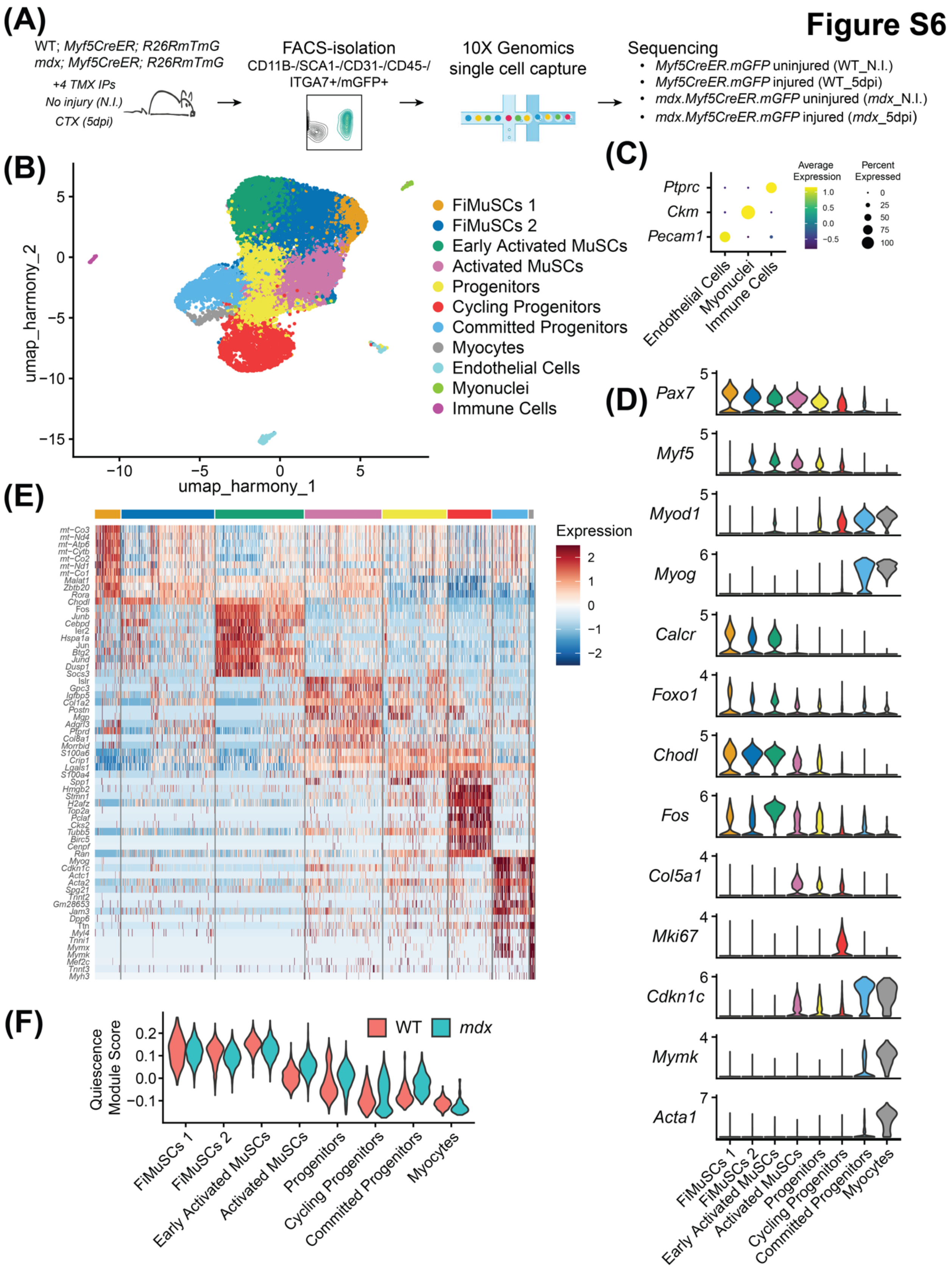
Figure S6
(A)
WT; Myf5CreER; R26RmTmG
mdx; Myf5CreER; R26RmTmG
+4 TMX IPs
No injury (N.I.)
CTX (5dpi)
FACS-isolation
CD11B-/SCA1-/CD31-/CD45-/ITGA7+/mGFP+
10X Genomics single cell capture
Sequencing
Myf5CreER.mGFP uninjured (WT_N.I.)
Myf5CreER.mGFP injured (WT_5dpi)
mdx.Myf5CreER.mGFP uninjured (mdx_N.I.)
mdx.Myf5CreER.mGFP injured (mdx_5dpi)
(B)
umap_harmony_1
umap_harmony_2
FiMuSCs 1
FiMuSCs 2
Early Activated MuSCs
Activated MuSCs
Progenitors
Cycling Progenitors
Committed Progenitors
Myocytes
Endothelial Cells
Myonuclei
Immune Cells
(C)
Ptprc
Ckm
Pecam1
Average Expression
Percent Expressed
Endothelial Cells
Myonuclei
Immune Cells
(D)
Pax7
Myf5
Myod1
Myog
Calcr
Foxo1
Chodl
Fos
Col5a1
Mki67
Cdkn1c
Mymk
Acta1
(E)
Expression
(F)
Quiescence Module Score
WT
mdx

Figure S6 (related to Figures 5 & 6). Sequencing of single myogenic cells identifies cell identity.

(A) Experimental design for fluorescence sorting and sequencing of single ITGA7$^+$ GFP$^+$ muscle stem cells (MuSCs) from uninjured (not injured, N.I.) and 5-day cardiotoxin-injured (5dpi) hindlimb muscle of WT and *mdx Myf5-Cre;RmTmG* mice. (B) Uniform manifold approximation and projection (UMAP) visualization of integrated uninjured and 5 dpi injured libraries (21,352 cells total) before subset and coloured by cluster identity. (C) Dotplot of contaminating cluster markers. (D) Violin plots of select identity markers for each cluster. (E) Heat map of top 10 differentially expressed genes per cluster. (F) Quiescence module score (top 400 enriched from Garcia-Prat et al. 2020[17]) across the myogenic clusters, divided between WT and *mdx* libraries.

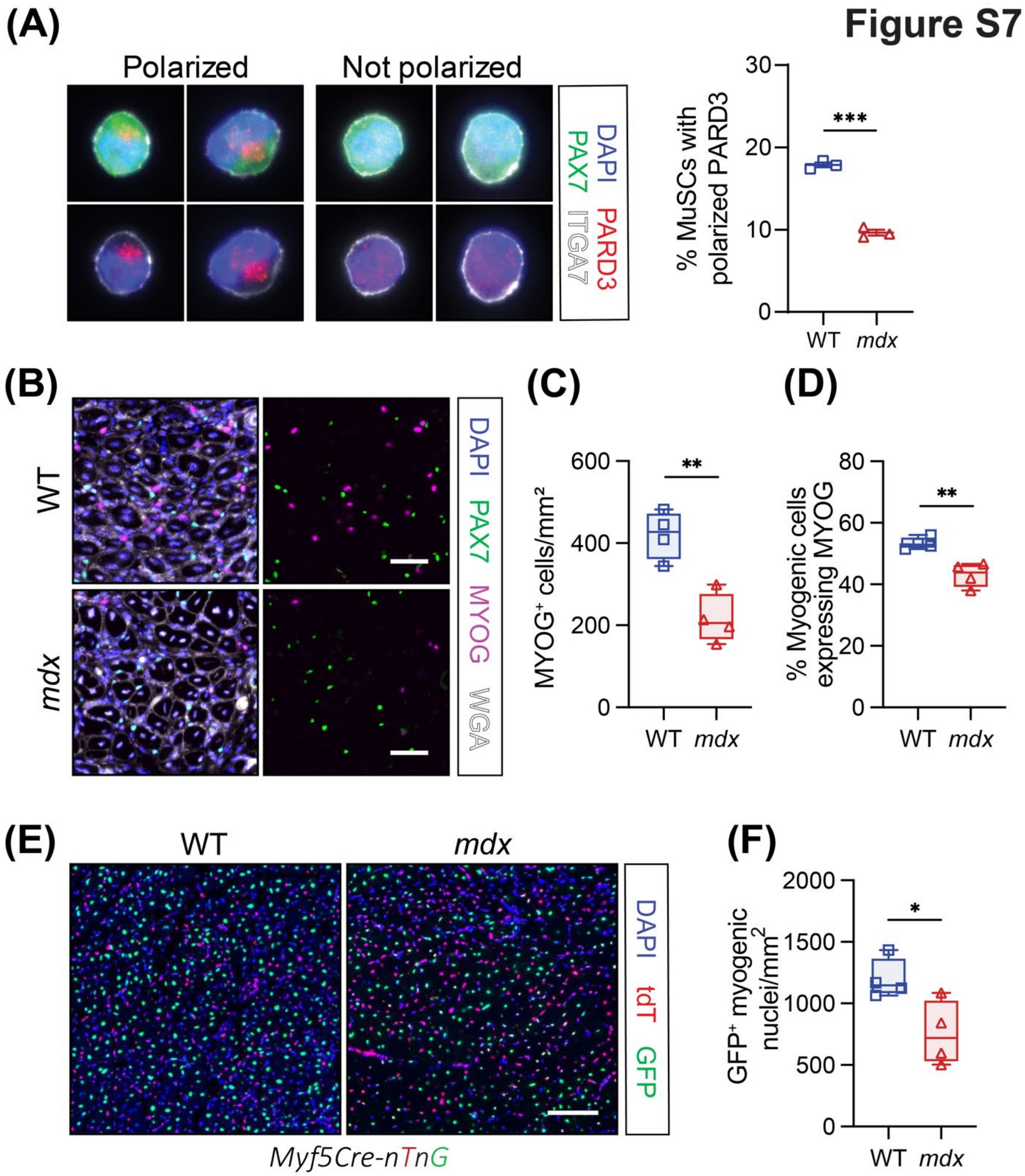

Figure S7. Impaired *mdx* MuSC polarity results in reduced generation of myocytes.

(A) Representative immunostaining of polarized and non-polarized PARD3 (red) MuSCs (PAX7, green; ITGA7, white) isolated 48 hours after cardiotoxin (CTX)-injury (left). Scale bar: 10µm. Quantification of the number of wild-type (WT) and *mdx* MuSCs with polarized PARD3 (right). n = 3 mice/genotype,

greater than 500-1000 cells quantified per condition. (B) Representative immunofluorescence image of WT and *mdx tibialis anterior* (TA) muscle transverse sections 5 days following CTX-injury (5 dpi). PAX7 (green) denotes the MuSCs, MYOG (magenta) stains the differentiated myogenic cells, WGA (white) labels the connective tissues and DAPI (blue) labels the nuclei. Scale bar: 50µm (C) Enumeration of MYOG-expressing ($MYOG^+$) cell number normalized to mm^2 on TA sections at 5 dpi. (D) $MYOG^+$ cell proportion relative to total $PAX7^+$ and $MYOG^+$ myogenic cells at 5 dpi. (E) Representative immunofluorescence images of WT and *mdx* TA cross-sections from WT and *mdx Myf5cre;ROSA-nTnG* mice at 7 dpi. Myogenic nuclei that transcribe the muscle regulatory factor *Myf5* express nuclear EGFP (green), while the remainder that never express *Myf5* express nuclear tdTomato (tdT, red). DAPI (blue) labels the nuclei. Scale bar: 200µm. (F) Quantification of WT and *mdx* GFP^+ myogenic nuclei at 7 dpi on TA cross-sections. Panel B-F, n = 4 mice/genotype. Boxplot whiskers represent maximum and minimum data values. Statistical analysis performed using unpaired *t*-tests where $*p < 0.05$; $**p < 0.01$; $***p < 0.001$.

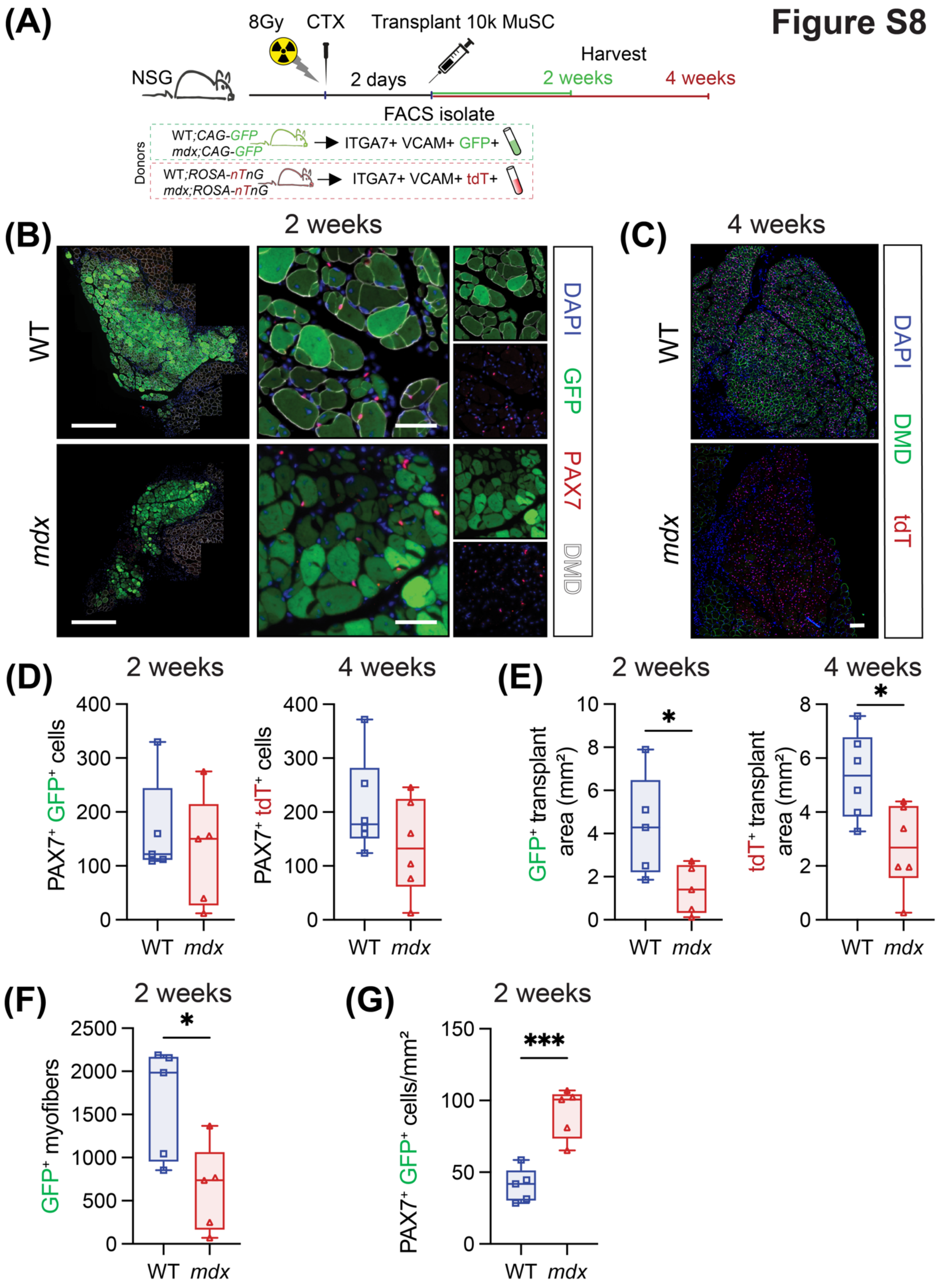
Figure S8
(A)
8Gy
CTX
Transplant 10k MuSC
Harvest
NSG
2 days
2 weeks
4 weeks
FACS isolate
Donors
WT;CAG-GFP
mdx;CAG-GFP
ITGA7+ VCAM+ GFP+
WT;ROSA-nTnG
mdx;ROSA-nTnG
ITGA7+ VCAM+ tdT+
(B)
2 weeks
WT
mdx
DAPI
GFP
PAX7
DMD
(C)
4 weeks
WT
mdx
DAPI
DMD
tdT
(D)
2 weeks
PAX7⁺ GFP⁺ cells
4 weeks
PAX7⁺ tdT⁺ cells
WT mdx
(E)
2 weeks
GFP⁺ transplant area (mm²)
4 weeks
tdT⁺ transplant area (mm²)
*
(F)
2 weeks
GFP⁺ myofibers
*
(G)
2 weeks
PAX7⁺ GFP⁺ cells/mm²
***

Figure S8 (related to Figure 8). Impaired muscle regeneration by *mdx* MuSCs.

(A) Experimental design for fluorescence sorting and injecting 10,000 donor ITGA7^{+}VCAM^{+} MuSCs from wild-type (WT) and *mdx CAG-GFP* (2-week harvest) or *ROSA-nTnG* (4-week harvest) mice into the contralateral *tibialis anterior* (TA) muscles of 2 days post-cardiotoxin (CTX) injured and irradiated NSG mice. For 2-week harvest, n = 5 mice/genotype; and 4-week harvest, n = 6 mice/genotype. (B) Immunostaining of host TA muscle cross-sections 2 weeks after the transplantation of MuSCs from WT and *mdx CAG-GFP* mice. Both MuSCs (PAX7, red) and myofibers (DMD, white) from the donors express GFP (green). DAPI stains the nuclei. Scale bars: left panels, 500µm; right panels, 50µm. (C) Immunostaining of host TA muscle 4 weeks after engraftment of WT and *mdx* MuSC from *ROSA-nTnG* mice. Donor myogenic nuclei express tdTomato (tdT, red). Dystrophin staining denotes WT myofibers (green) and DAPI labels nuclei (blue). Scale bars are all 100µm. (D) Quantification of donor PAX7^{+} cells on TA cross-sections at 2 weeks (GFP^{+}, left) and 4 weeks (tdT^{+}, right) following MuSC transplantation. (E) Transplant area 2 weeks (left) and 4 weeks (right) following donor MuSC injection. (F) Number of engrafted GFP^{+} myofibers enumerated 2 weeks after transplantation. (G) PAX7^{+}/GFP^{+} MuSC number per mm^2 two weeks after engraftment. Boxplot whiskers represent maximum and minimum data values. Statistical analysis performed using unpaired *t*-tests where $*p < 0.05$; $**p < 0.01$; $***p < 0.001$.

Supplemental tables

Table S1. List of antibodies

Primary Antibody	Company	Reference	Dilution
Mouse anti-PAX7	DSHB	Ab 528428;	1/2
Mouse anti-MYOGENIN (clone F5D)	Santa Cruz	sc-12732	1/500
Mouse anti-DYSTROPHIN	Leica	NCL-DYS1	1/5
Rabbit anti-DYSTROPHIN	Abcam	ab15277;	1/1000
Rat anti-LAMININ [4H8-2]	Sigma-Aldrich	L0663	1/1000
Rabbit anti-LAMININ	Sigma	L9393	1/500
Rabbit anti-COLLAGEN I (COL 1)	Abcam	ab21286	1/500
Rabbit anti-PARD3	Millipore	07330	1/500
Chicken anti-GFP	Abcam	ab13970	1/1000
Alexa647 mouse anti-Integrin alpha7 (clone R2F2)	UBC AbLab	67-0010-10	1/100
Mouse anti-Integrin alpha7-Biotin (clone 3C12)	Miltenyi Biotec	130-102-125	1/100
Satellite Cell Isolation Kit	Miltenyi Biotec	130-104-268	1/100
BV421 mouse anti-SCA1 (clone D7)	BD Biosciences	553108	1/500
BV421 mouse anti-CD45 (clone 30-F11)	BD Biosciences	12-0451-83	1/500
BV421 mouse anti-CD31 (clone 390)	BD Biosciences	12-0311-81	1/500
BV421 mouse anti-CD11b (clone M1/70)	BD Biosciences	12-0112-81	1/500
PE-Cy7 mouse anti-CD106 (VCAM1)	BioLegend	105719	1/100
PerCP/Cyanine5.5 Streptavidin	BioLegend	405214	1/500

Table S2. Other reagents

Primary Antibody	Company	Reference
Wheat Germ Agglutinin Alexa 647 conjugate	Fisher	W32466
7-AAD (7-Aminoactinomycin D)	STEMCELL Technologies	75001.1
DAPI (4',6-Diamidino-2-Phenylindole,Dihydrochloride)	Thermo Fisher Scientific	D1306
bisBenzimide Hoechst 33342 trihydrochloride	Sigma-Aldrich	B2261
M.O.M. (Mouse on Mouse) Blocking Reagent	Vector Laboratories	VECTMKB22131
Tamoxifen	Sigma-Aldrich	T5648
Cardiotoxin	Latoxan	L8102
PicoPure™ RNA Isolation Kit	Applied Biosystems	KIT0204
Formaldehyde Aqueous Solution	Electron Microscopy Sciences	157-8
Corning™ Cell-Tak Cell and Tissue Adhesive	Thermo Fisher Scientific	CB40240
